# Selective Stepwise Reduction of Nitrate and Nitrite to Dinitrogen or Ammonia

**DOI:** 10.1021/jacs.4c16585

**Published:** 2025-02-28

**Authors:** Jewelianna M. Moore, Tabitha J. Miller, Manting Mu, Marconi N. Peñas-Defrutos, Kelly L. Gullett, Lindsey S. Elford, Sebastian Quintero, Max García-Melchor, Alison R. Fout

**Affiliations:** † Department of Chemistry, Texas A&M University, 580 Ross St., College Station, Texas 77843, United States; ‡ School of Chemical Sciences, University of Illinois at Urbana−Champaign, Urbana, Illinois 61801, United States; § School of Chemistry, CRANN and AMBER Research Centres Trinity College Dublin, College Green, Dublin 2 Dublin 2, Ireland; ∥ IU CINQUIMA, Química Inorgánica, Facultad de Ciencias, Universidad de Valladolid, 47071 Valladolid, Spain; ⊥ Center for Cooperative Research on Alternative Energy (CIC EnergiGUNE), Basque Research and Technology Alliance (BRTA), Alava Technology Park, Albert Einstein 48, 01510 Vitoria-Gasteiz, Spain; # IKERBASQUE, Basque Foundation for Science, Plaza de Euskadi 5, 48009 Bilbao, Spain

## Abstract

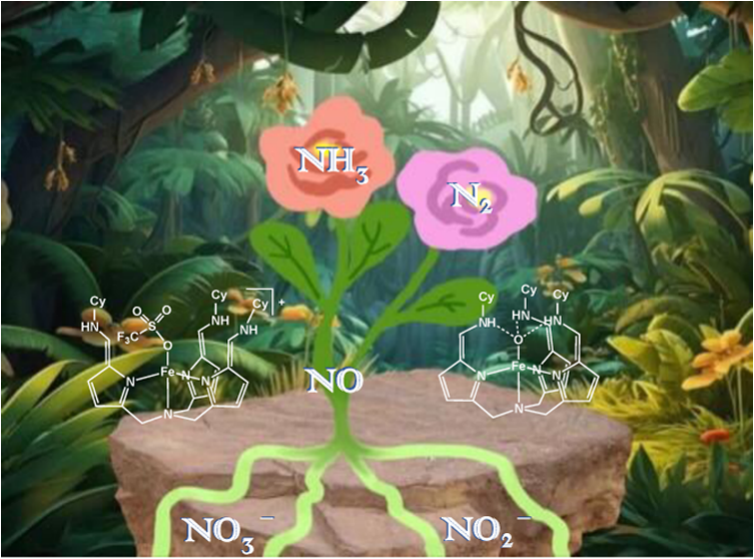

This study reports a method for the selective reduction of NO_3_^–^ and NO_2_^–^ to N_2_ or NH_3_, extending prior work in our lab where NO_3_^–^ was reduced to NO by [N(afa^Cy^)_3_Fe]OTf_2_ (N(afa^Cy^)_3_ = tris(5-cyclohexyl-amineazafulvene-2-methyl)amine, OTf = triflate). The first pathway involves the reduction of NO_2_^–^ to N_2_, where the NO generated in the initial step is transformed to N_2_O by PPh_3_ and further reduced to N_2_ by the [N(afa^Cy^)_3_Fe]OTf_2_ complex. An alternative pathway showcases the reduction of the bound NO complex, [N(afa^Cy^)_3_Fe(NO)]^2+^, to NH_3_ using chemical reductants, albeit with a modest yield of 29%. Confirmation of the nitrogen source as NO is established through ^15^N labeling studies. Hydroxylamine (NH_2_OH) is proposed as a plausible intermediate in the reduction of bound NO, supported by independent NH_2_OH reduction experiments and computational studies. Nature employs a well-orchestrated, stepwise process involving several enzymes to reduce N-containing oxyanions, and this approach provides valuable insights into the stepwise reduction mechanisms of nitrate and nitrite, yielding NH_3_ or N_2_ as the product.

## Introduction

**Scheme 1:**
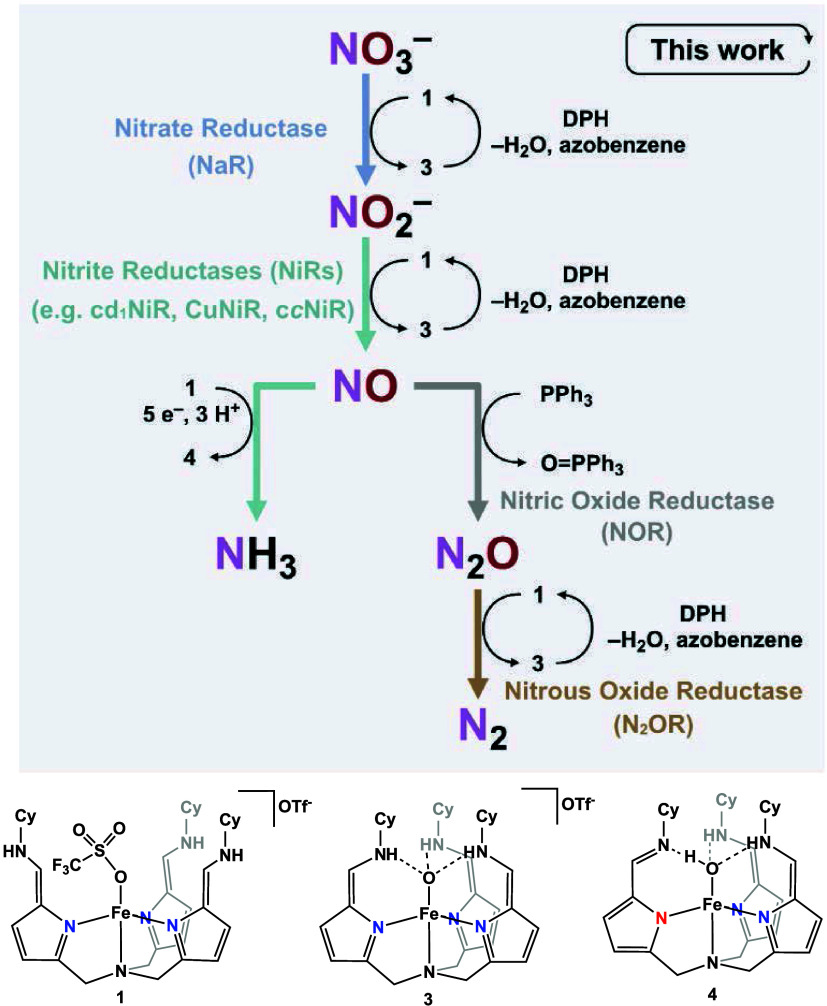
Stepwise Reduction of NO_3_^–^ to NH_3_ (Left) or N_2_ (Right) by Nature and by our Synthetic Complex [N(afa^Cy^)_3_Fe]OTf_2_ (**1**). The Arrows Representing the Natural Pathways are Color-coded to Correspond to the Appropriate Enzymes: Nitrate Reductase (NaR), Cytochrome *cd_1_* Nitrite Reductase (cd_1_NiR), Nitric Oxide Reductase (NOR), Nitrous Oxide Reductase (N_2_OR), and Cytochrome *c* Ntrite Rductase (ccNiR). The Bottom Panel shows the Structure of Compound **1** Along with the Isolated Reaction Intermediates (**3** and **4**) Formed During the Sepwise Reduction Process.

Nitrate (NO_3_^–^) and nitrite (NO_2_^–^), common water pollutants,^1,2^ play pivotal roles in the global nitrogen cycle, acting as terminal electron acceptors in various biochemical processes. To reduce these N-containing compounds, Nature has evolved finely tuned, stepwise enzymatic processes. Two primary pathways exist for nitrate reduction, leading to the formation of either ammonium (NH_4_^+^) or dinitrogen (N_2_), but not both.^3,4^ In the denitrification pathway (Scheme 1, right) NO_3_^–^ is reduced to N_2_ through a multistep process carried out by a series of enzymes. Nitrate is initially reduced to nitrite by the molybdenum-dependent nitrate reductase (NaR),^4,5^ followed by nitrite reduction to nitric oxide (NO) via heme-based nitrite reductase (cd_1_NiR)^3,6^ or copper-based nitrite reductase (CuNiR).^3,6,7^ The critical formation of the N–N bond occurs when NO is reduced to nitrous oxide (N_2_O) by either heme or nonheme nitric oxide reductase (NOR).^8,9^ The final step, catalyzed by iron/copper-containing nitrous oxide reductase (N_2_OR), reduces N_2_O to N_2_.^10^

On the other hand, in the dissimilatory nitrate reduction (DNRA) pathway (Scheme 1, left), NO_3_^–^ is first deoxygenated to NO_2_^–^ by NaR.^4^ Subsequently, the heme-containing cytochrome *c* nitrite reductase (ccNiR) reduces NO_2_^–^ directly to NH_4_^+^,^3,11^ which is then incorporated into the organic nitrogen pool, contributing to the production of amino acids such as glutamate and glutamine.^12^ This intricate interplay of enzymes highlights the sophistication of Nature’s nitrogen cycling mechanisms.

Human activities, particularly the persistent use of fertilizers, have disrupted the natural nitrogen cycle, leading to adverse effects such as eutrophication and contamination of groundwater and drinking water.^2,13,14^ To address these environmental challenges, the conversion of NO_x_^–^ compounds has become crucial in remediation efforts and has driven the exploration of synthetic systems capable of reducing N-containing oxyanions. While biological systems efficiently and selectively reduce NO_3_^–^ to specific N-containing products, synthetic systems typically struggle to achieve high selectivity. Most synthetic systems involve solely deoxygenation events, reducing nitrate or nitrite to nitric oxide, resembling the incomplete reduction of nitrate observed in Nature (Scheme 1).^15−44^ This underscores the ongoing challenge of developing synthetic systems that can achieve the selectivity and efficiency of the natural nitrogen reduction processes.

The electrochemical reduction of nitrate^45−49^ and nitrite^45,48,50−56^ to ammonia or ammonium has been extensively studied.^57−60^ However, synthetic systems have been lesser explored, with only a few synthetic systems capable of one of the two reduction pathways observed in Nature. Specifically, two synthetic systems have demonstrated the ability to reduce NO_2_^–^ to NH_3_ but remain incapable of reducing NO_3_^–^ as seen in the DNRA pathway.^61,62^ In contrast, denitrification pathways have been more successfully modeled by synthetic systems. In 2019, Lee and co-workers demonstrated complete denitrification using a single metal site.^63^ Their (PNP)nickel(II) pincer complex efficiently reduced NO_3_^–^ to N_2_ gas, employing carbon monoxide as the oxygen atom acceptor to produce carbon dioxide. A year later, Gilbertson and co-workers developed a system using Samarium(II) iodide that could stoichiometrically reduce NO_3_^–^ to N_2_.^64^ Despite these advances, to the best of our knowledge, there is currently no known molecular system capable of selectively reducing NO_3_^–^ to either NH_3_ or N_2_ gas depending on the conditions of the system. Drawing inspiration from Nature, we propose the production of a synthetic system which models the stepwise reductions like those observed in the DNRA and denitrification pathways, following the N-containing intermediates identified or hypothesized in each pathway.

**Scheme 2:**
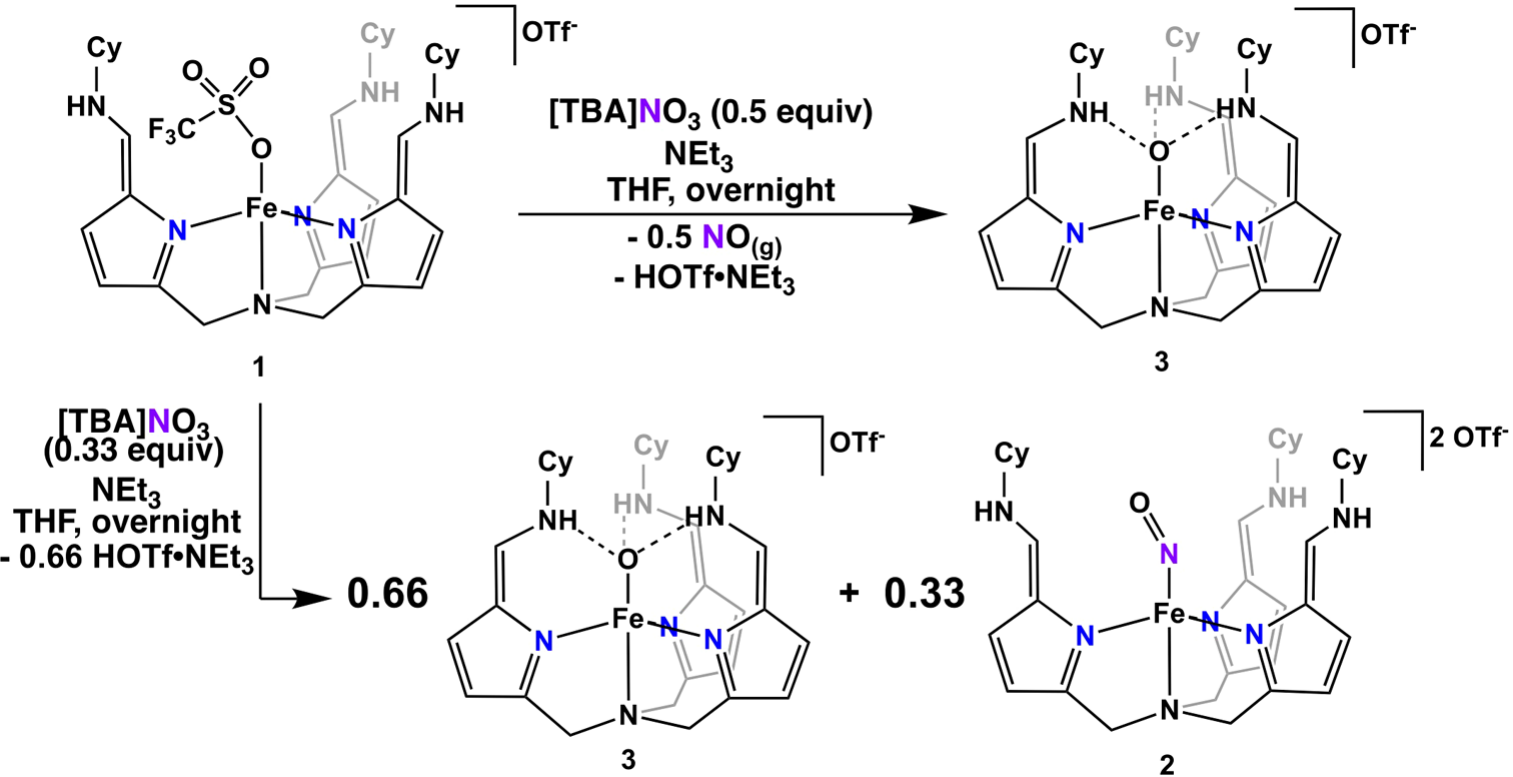
Reduction of [TBA]NO_3_ by Complex**1** to Complex**3** and NO Gas (top) or Complexes **3** and **2** (Bottom) Dependent on the Number of Equivalents of [TBA]NO_3_ Used

Herein we report a distinctive and selective process for the stepwise reduction of NO_3_^–^ and NO_2_^–^ to either N_2_ or NH_3_. This is achieved using a tripodal iron complex in conjunction with a sacrificial reductant, effectively emulating the denitrification and dissimilatory nitrate reduction pathways observed in Nature. The tripodal ligand framework, tris(5-cycloimminopyrrol-2-ylmethyl)amine ([N(pi^Cy^)_3_]), previously reported, is capable of tautomerization and features H-bond donating and accepting moieties in the secondary coordination sphere.^65^ Our prior research utilizing this framework highlighted the catalytic reduction of NO_3_^–^ or NO_2_^–^ to NO using the metalated iron complex [N(afa^Cy^)_3_Fe]OTf_2_ (**1**).^15,16^ During the catalytic reduction, the formation of a terminal Fe(III)-oxo species, [N(afa^Cy^)Fe^III^(O)]^+^ (**3**), and NO gas were observed (Scheme 2, top). Compound **3** was then turned over with 1,2-diphenylhydrazine to reform **1**, producing an equivalent of water and azobenzene, thereby making the reduction catalytic. Unlike ccNiR, **1** is unable to further deoxygenate or reduce NO, which binds to **1** forming a stable iron-nitrosyl complex, [N(afa^Cy^)Fe(NO)]^2+^ (**2**) (Scheme 2, bottom).^15,16^ Given the environmental significance of NO, which contributes to acid rain and reacts with ozone to produce N_2_O, a potent greenhouse gas,^66^ the focus of this work is on the selective reduction of NO to either N_2_ or NH_3_. This approach mimics the NO_x_^–^ (x = 2, 3) reduction pathways observed in Nature and aims to inform the development of synthetic systems for sustainable N_2_ and NH_3_ production from NO_x_^–^.

## Results and Discussion

### Nitrate/Nitrite Reduction to Dinitrogen

**Scheme 3:**
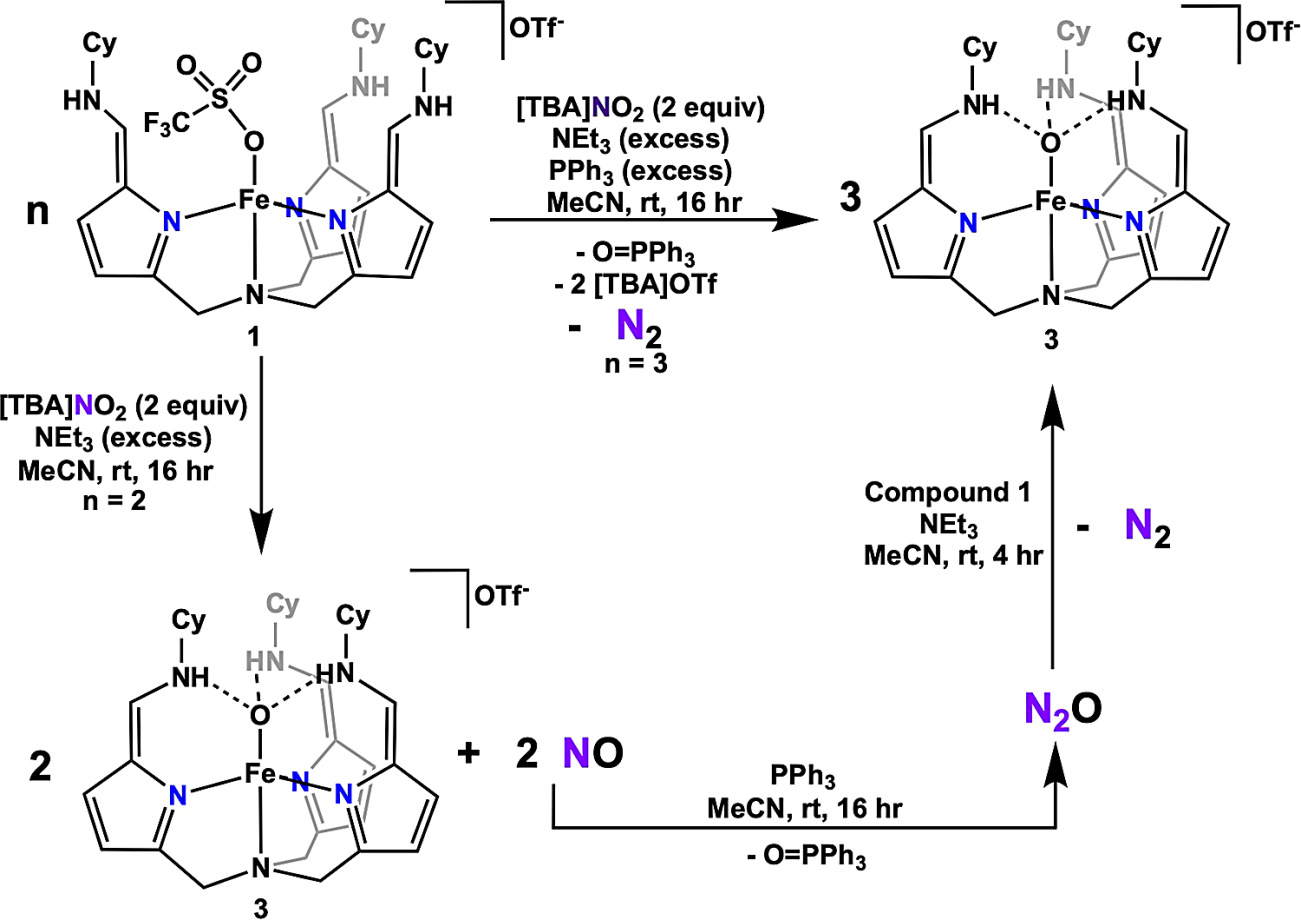
Stepwise Reduction of Nitrite to N_2_ by Complex**1** and PPh_3_. The Top Pathway Shows a Summary of the Overall Reaction. The Bottom Pathway Shows the Stepwise Reactions.

To model the denitrification pathway, a one-pot reduction of NO_3_^–^ or NO_2_^–^ to N_2_O was carried out, followed by the reduction of N_2_O to N_2_ (Scheme 3). In the former reaction, compound **1** reduced NO_3_^–^ or NO_2_^–^ (2 or 1 equiv of compound **1** was used respectively), yielding compound **3** (2 or 1 equiv) and releasing one equivalent of NO gas. To further reduce the NO gas produced during this process, triphenylphosphine (PPh_3_) was utilized as a mild external reductant, known to convert NO to N_2_O, while forming triphenylphosphine oxide (O=PPh_3_).^67^

In the one-pot reduction of nitrite to nitrous oxide, a reaction mixture containing one equivalent of compound **1** and tetrabutylammonium nitrite ([TBA]NO_2_), along with an excess of PPh_3_, was stirred overnight (Scheme 3). After extracting the phosphine product(s) with ether, ^31^P NMR spectroscopy confirmed the clean formation of triphenylphosphine oxide. Using triphenylphosphine sulfide as an internal standard, the yield of O=PPh_3_ was quantified by ^31^P NMR spectroscopy, achieving quantitative yield based on integration. To rule out the possibility of deoxygenation of the iron-oxo species, compound **3** was stirred with PPh_3_ in acetonitrile overnight. After workup, no O=PPh_3_ was detected by ^31^P NMR spectroscopy. Additionally, no reaction was observed between compound **2** and PPh_3_, indicating that nitric oxide must remain unbound to the iron center for effective reduction. Therefore, this reaction must take place in two steps to preclude the formation of complex **2** prior to reduction of NO by PPh_3_.

In this reduction of nitrite to nitrous oxide, compound **3** was identified as the sole Fe-containing product by ^1^H NMR spectroscopy and was isolated in quantitative yield. Gas chromatography head space analysis confirmed nitrous oxide was the only gas produced, with no detectable nitric oxide, demonstrating the role of PPh_3_ as a sacrificial reductant in the reduction of NO generated *in situ* from nitrite.

Although the reduction of N_2_O to N_2_ is thermodynamically favorable, nitrous oxide is typically kinetically inert, requiring high temperatures and pressures to oxidize most organic compounds.^68−70^ However, there are precedents for the reduction of nitrous oxide to dinitrogen by metal complexes under ambient or low-temperature conditions.^68,69,71−76^ Given the success of **1** in reducing kinetically inert oxyanions such as perchlorate, and forming a stable iron-oxo species **3**,^77,78^ we proposed the reduction of the generated N_2_O by compound **1** (Scheme 3). The reaction of **1** with N_2_O under inert atmosphere led to the formation of **3**, as confirmed by ^1^H NMR spectroscopy. Initial experiments produced proto-demetalated ligand (N(pi^Cy^)_3_·3OTf) along with **3**, similar to the reactivity of **1** with dioxygen.^79^ To suppress proto-demetalated ligand formation, triethylamine was added to the reaction mixture, successfully leading to the exclusive formation of **3**, as confirmed via ^1^H NMR spectroscopy. Based on similar metal-based systems where N_2_O serves as an oxygen atom transfer reagent,^69,71−76^ we propose the formation of dinitrogen gas as the N-containing product, thus illustrating the stepwise reduction of nitrate or nitrite to nitric oxide to nitrous oxide in situ, followed by the reduction of nitrous oxide to dinitrogen.

### Nitrate Reduction to Ammonia

To model the dissimilatory nitrate reduction pathway, we investigated the reduction of the bound nitrosyl in **2** formed in nitrate reduction to ammonia, focusing on the novel reduction step. Initial trials with mild to moderate reductants, such as 1,2-diphenylhydrazine (DPH), sodium naphthalenide, and cobaltocene, showed no reaction with **2**. However, introducing potent chemical reductants, such as potassium graphite (KC_8_) or sodium potassium alloy (NaK), led to the disappearance of the nitrosyl complex **2** and the concomitant formation of the previously reported hydroxide complex, N(afa^Cy^)_2_(pi^Cy^)FeOH (**4**),^65^ as confirmed by ^1^H NMR spectroscopy. Based on these findings, we envisioned a mechanism where NO is reduced to form NH_3_ and H_2_O, analogous to the process observed in ccNiR, with the resulting water binding to the iron center, forming the metal-bound OH compound **4**.^53^

**Figure 1:**
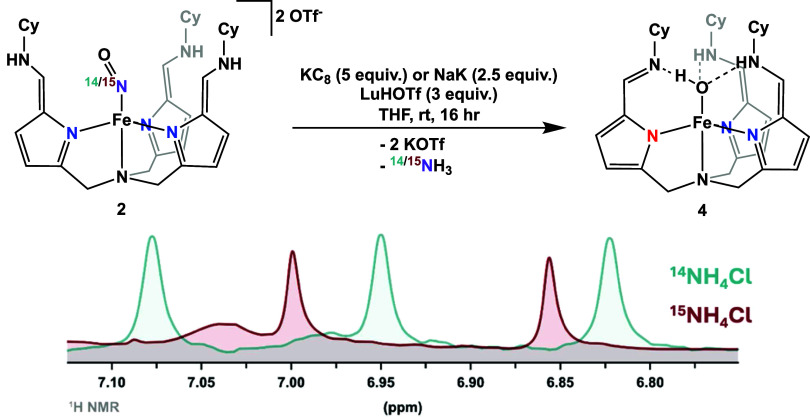
Top: reduction of complex **2 or^15^N-2** to complex **4** and ammonia. Bottom: selected region of the ^1^H NMR spectra of the acid traps, displaying signals for the formation of ^14^NH_4_Cl (blue, ^1^J_N–H_ = 51 Hz) and ^15^NH_4_Cl (red, ^1^J_N–H_ = 71 Hz) in DMSO-*d*_6_.

To optimize the system for the 5e^–^/5H^+^ reduction of NO to NH_3_ and **4**, five electron equivalents of the reductant (5 equiv KC_8_ or 2.5 equiv NaK) and three equivalents of 2,6-lutidinium triflate (LuHOTf) were added to a THF solution of complex **2** at room temperature (Figure 1, top), with the ligand providing two additional proton equivalents during the process, facilitating its completion.

While the addition of KC_8_ and LuHOTf to previously published nitrate reduction conditions (Scheme 2, bottom) resulted in the formation of ammonia, we focus on the direct reduction of complex **2** to gain insight into the novel reaction steps. The addition of KC_8_ (5 equiv) or NaK (2.5 equiv) and LuHOTf (3 equiv) to **2** resulted in the formation of **4** in moderate crystalline yield (56.7%). To confirm the formation of NH_3_, an acid trap was employed to convert the generated NH_3_ to NH_4_Cl, which was identified by ^1^H NMR spectroscopy. The NH_4_^+^ proton peak exhibited the expected 1:1:1 triplet due to the coupling with the ^14^N nucleus (Figure 1, blue trace). The yield of ammonia, quantified by the indophenol method,^80^ was low to moderate depending on the reductant used (9.5% for KC_8_ and 28.7% for NaK). Initially, the low ammonia yield was attributed to poor selectivity toward any N-containing product. However, gas chromatography head space analysis confirmed ammonia as the sole N-containing gas, demonstrating the selectivity of the system. Nevertheless, a substantial amount of H_2_ gas was also produced due to the use of a strong reductant in conjunction with acid which likely is limiting the overall yield of ammonia produced.

To confirm that NH_3_ formation arises from the reduction of the bound nitrosyl rather than from ligand-based nitrogen atoms, control reductions of ligand (N(pi^Cy^)_3_) and complex **1** were conducted under the same conditions described above using NaK. The ammonia yields, as determined by the indophenol method, were significantly lower at 4.1 and 1.8%, respectively, compared to the reduction of **2** (28.7%). To further support this finding, ^15^N-labeled ^15^NO_2_^–^ was used to ascertain that the ammonia produced originated from the reduction of the coordinated nitrosyl, rather than from the decomposition of metal complex **2** or the free ligand.

The ^15^N-labeled nitrosyl complex, [N(afa^Cy^)_3_Fe^15^NO]OTf_2_ (^**15**^**N-2**), was generated by the reduction of Na^15^NO_2_ by **1** (2 equiv), followed by separation of ^**15**^**N-2** from the equivalent amount of **3** produced (see SI for details). ^**15**^**N-2** exhibited an identical ^1^H NMR spectrum to the unlabeled complex **2** and displayed the expected shift of the N = O stretching frequency from ν_NO_ = 1717 cm^–1^ to ν_15NO_ = 1744 cm^–1^ for the ^15^N isotopologue in the infrared spectrum.^15^ Subsequently, the reduction of ^**15**^**N-2** was carried out under the same conditions as **2** with an acid trap. The trapped NH_4_Cl salt was identified as ^15^NH_4_Cl by ^1^H NMR spectroscopy, showcasing the expected splitting pattern of a doublet and an N–H coupling value of 71 Hz (Figure 1, red trace). Notably, the triplet of ^14^NH_4_Cl was absent in the ^1^H NMR spectrum, provides clear confirmation that the generated ammonia results from the reduction of the bound nitrosyl in **2**.

### Hydroxylamine Reduction to Ammonia

Given that the reduction of **2** to ammonia requires strong heterogeneous chemical reductants, conventional methods for mechanistic studies, such as UV–visible, NMR, and react infrared spectroscopies are not applicable to this system. Therefore, to gain mechanistic insight, we explored the reduction of hydroxylamine to ammonia by **1**, as hydroxylamine is a commonly proposed intermediate in the reduction of nitrite to ammonia in ccNiR and related inorganic systems.^81,82^ Specifically, ccNiR is known to reduce NO_2_^–^, NO, and NH_2_OH to NH_3_ without the release of detectable intermediates. Additionally, NH_2_OH has been crystallized in the active site of ccNiR, suggesting its viability as an intermediate in nitrite reduction.^82^

**Scheme 4:**
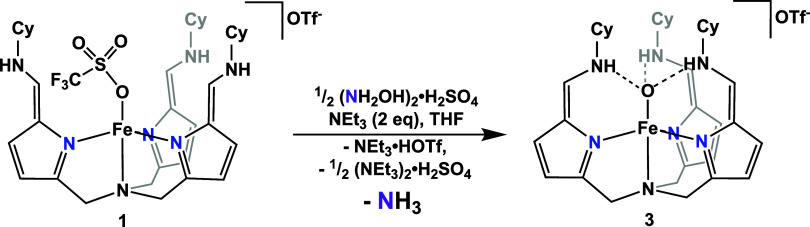
Reduction of Hydroxylamine by Complex**1** to Form Complex**3** and Ammonia

In contrast to the reduction of **2**, the reduction of hydroxylamine to ammonia by **1** proceeds without the need for external reductant (Scheme 4). When [(NH_2_OH)_2_]H_2_SO_4_ is subjected to **1** (2 equiv), **3** is observed via ^1^H NMR spectroscopy and is isolated in high yield (70.6% crystalline yield). In the absence of a proton source or external reducing agents, the anticipated product of the 2e^–^/2H^+^ reduction of NH_2_OH would be an iron(IV)-oxo if the generated H_2_O were to bind to the metal center. However, we propose that high valent iron species are unstable in this tripodal ligand framework and instead hypothesize that an H atom is abstracted, forming an iron(III)-hydroxide, [N(afa^Cy^)_3_FeOH]OTf_2_. Our prior work demonstrates that this complex can be cleanly deprotonated to form **3**.^79^ The formation of ammonia was confirmed using an acid trap, and the yield, though moderate, of 29.8%, as assayed by the indophenol method, aligns well with the ammonia yield obtained in the reduction of **2** using NaK. While the system’s ability to reduce hydroxylamine suggests that it could be a viable intermediate in the reduction of nitrate to ammonia, we turned to computational methods to gain additional mechanistic insight.

### Mechanistic Insights on NO to NH_3_ Reduction

To shed light on the mechanism of NO reduction to NH_3_, we performed density functional theory (DFT) calculations on the NO-bound iron complex **2** in THF under experimental conditions (see Figure 1). The calculations were carried out using Gaussian 16 software at the ωB97XD level of theory, considering key ligand conformers and high-spin states of iron metal (ferro- and antiferromagnetically coupled spin states), focusing on those with the lowest energy. The proton-coupled electron transfer (PCET) steps involved in the reaction mechanism were modeled using the method reported by Van Voorhis et al.,^83^ and considering the redox potential of the KC_8_ reductant (see SI for details). Additionally, the effect of solvent THF, including its H-bond acceptor capability, was accounted for through the continuum solvation model (SMD), adding a correction factor derived from our previous work.^84^

**Figure 2:**
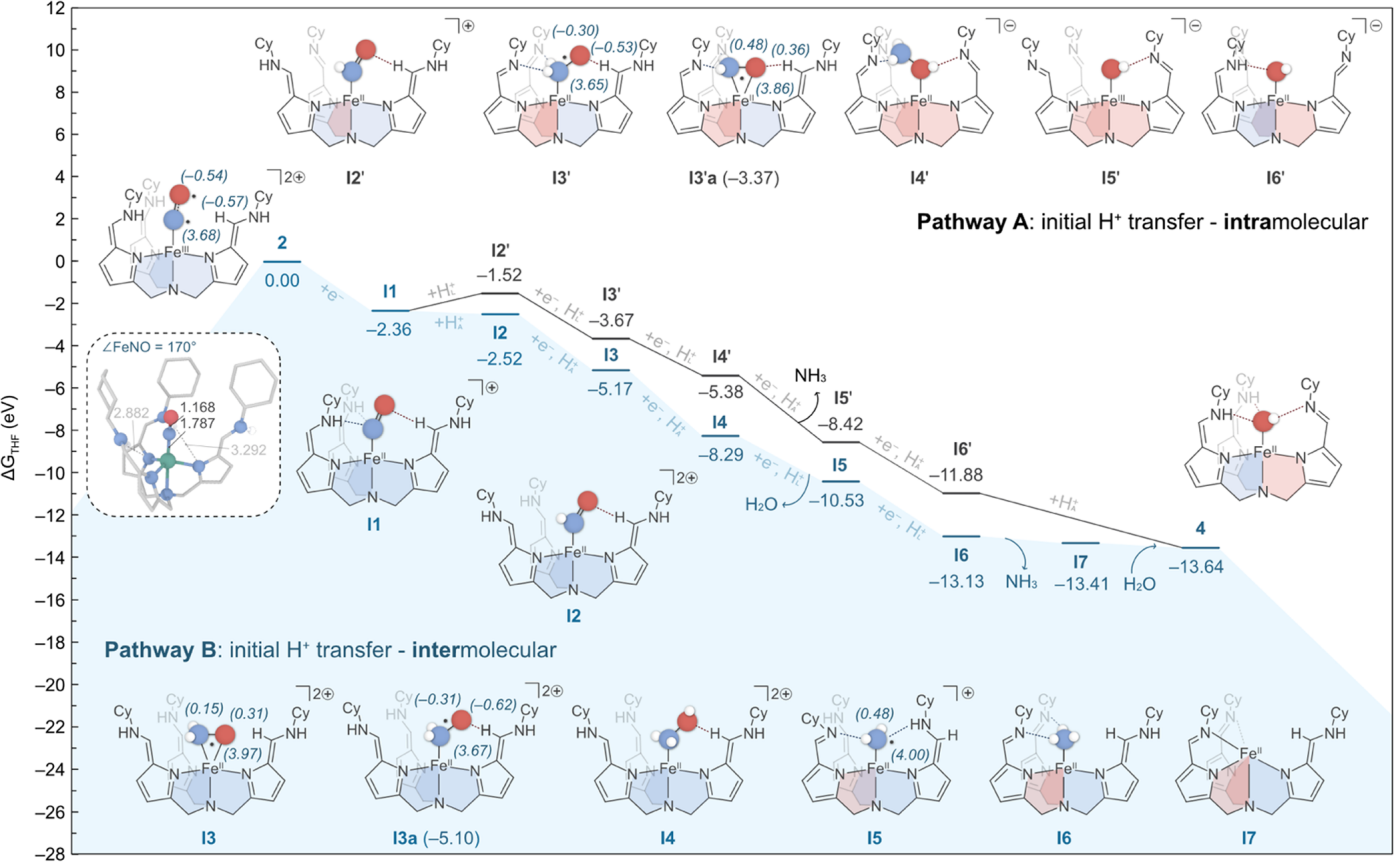
Gibbs energy profile calculated in THF at 298 K and 1 atm for the reduction of complex **2** to **4** and NH_3_ with KC_8_ serving as the sacrificial reducing agent (e^–^), providing electrons, while LuHOTf acts as the external proton source (H_A_^+^), the tripodal ligand offers the internal proton source (H_L_^+^). The two lowest-energy pathways in eV (1 eV = 23.06 kcal/mol) are shown, initiated by either intramolecular (pathway A, black) or intermolecular (pathway B, blue) proton transfer steps. The N(afa^Cy^) and N(pi^Cy^) ligand arms are shaded in blue and red, respectively to demonstrate the different ligand tautomeric forms. Spin densities (in a.u.) are shown in brackets for key radical species. The DFT-optimized structure of **2** is shown as an inset, with relevant bond distances provided in Å.

In our mechanistic investigation we propose that the NO in complex **2** is reduced through a series of PCET steps, which are commonly accepted in literature for similar electrocatalytic processes.^85^ In our work, the proton source for these steps could originate externally from the added acid (LuHOTf) or sourced internally from the N–H bond within the ligand, similar to the mechanism proposed for ccNiR, where amino acids serve as proton donors.^82,86^ The sequence in which protons are transferred onto the bound NO, either through the external acid or internal transfers, can vary, offering a broad range of possibilities. Herein we focused on two extreme scenarios to gain an overall understanding of the system’s reactivity. The first scenario assumes that all three initial protons are transferred from LuHOTf, followed by intramolecular transfers (pathway A, Figure 2, black trace), while the second assumes that all initial protons are supplied by intramolecular transfers within the ligand, followed by the addition of three external protons from LuHOTf (pathway B, Figure 2, blue trace). While alternative mechanisms for paths A and B were also considered (see Schemes S2 and S3), we focus here on describing the most energetically favorable pathways from the two extremes, shown in Figure 2.

We began our theoretical studies by modeling the NO-bound complex **2**, considering all possible conformers resulting from the rotation of the N(afa^Cy^) ligand arms, as shown in Scheme S1. The most favorable conformer features two arms with both nitrogen atoms in the same ligand arm in *cis* position. Notably, these *cis*-positioned arms do not orient their NH units toward the Fe-bound NO. This behavior is due to the Fe–N–O bond angle being 170°, and the lone pair of the N_NO_ which is primarily involved in bonding with Fe, making it unavailable for further interaction (see Figure 2, inset).

The DFT-optimized structure of complex **2** in the quartet spin state exhibits Mulliken spin densities of +3.68, −0.57, and −0.54 au on the Fe, N, and O atoms, respectively. We also examined the ferromagnetic sextet spin state, which yielded spin densities of +3.72, +0.65, and +0.31 au on the same atoms. Notably, the Fe–N bond length in the sextet (2.340 Å) is noticeably longer than in the quartet (1.787 Å), with the latter closely aligning with the values observed in similar Fe–NO complexes bearing tripodal ligand scaffolds.^87^ The X-ray crystallographic structure of complex **2** clearly reveals a [N(afa^Cy^)_3_FeNO]OTf_2_ unit featuring a nonlinear NO group; however, the data’s limited resolution restricts detailed assessment of bond lengths, including the Fe–N distance (Figure S17). Overall, the quartet spin state is more stable than the sextet by +0.33 eV, consistent with our previously reported 4K EPR spectrum, which indicate an *S* = 3/2 system.^15^

The EPR spectrum also suggests an Fe(III)–NO^–^ species with a triplet NO^–^, consistent with descriptions by Borovik et al.^87^ and proposed by Wieghardt and co-workers for Tp*M(NO) complexes (Tp* = hydro-tris(3,5-Me_2_-pyrazolyl)borate; M = Co, Ni).^88^ Our calculated Mulliken spin density on NO (−1.11 au) in the lowest-energy quartet spin state, more negative than −1.00, suggests that this moiety is best described as an NO^–^ with diradical character, antiferromagnetically coupled to the unpaired electrons on the Fe center. To further examine the Fe oxidation state and electronic structure, we performed CASSCF(7,7) calculations and CASCI(7,7) analysis on localized molecular orbitals derived from CASSCF wave functions for complex **2** (see SI for details).^89^ The CASCI analysis reveals a dominant configuration, with a 23.09% weight, corresponding to a singly antiferromagnetically coupled Fe(III)(*S* = 3/2)–NO^–^(*S* = 1) ground state configuration. Multireference analysis indicates that an Fe(III) oxidation state contributes approximately 90.15% to the ground state, with 82.75% in the intermediate-spin state (*S* = 3/2) and 7.58% in the high-spin state (*S* = 5/2). The Fe(III) spins show variable coupling with NO^–^, either singly, doubly, or uncoupled. Among these, NO^–^ exhibits 55.56% diradical character (*S* = 1) and 34.59% closed-shell singlet (*S* = 0) character. In summary, the electronic structure of compound **2** is complex, involving multiple spin coupling configurations, and is most accurately described as an Fe(III)(*S* = 3/2)–NO^–^(*S* = 1) system based on CASCI analysis.

Similar to the behavior of ccNiR, complex **2** undergoes a 1 e^–^ reduction to form the Fe(II)-bound {NO}^−^ intermediate (**I1**). Unlike complex **2**, in intermediate **I1**, the Fe–N–O bond angle decreases from 170 to 155°, suggesting that the lone pair on the N_NO_ is available for interaction. Indeed, in this intermediate, the NH units of the two *cis*-positioned ligand arms are oriented toward the Fe-bound NO, forming two hydrogen-bonding interactions with *r*(N_NO_–H_afacy_) = 1.880 and 1.944 Å. This step, common to both pathways A and B, is exergonic by −2.36 eV and is characterized by the quenching of the NO radical, accompanied by a tilting and elongation of the Fe–N_NO_ bond from 1.787 Å (Fe^2+^) to 1.965 Å (Fe^2+^). Once **I1** is formed, a proton transfer step yields an intermediate with a {NHO} moiety bound to Fe. When the proton is sourced from the ligand (pathway A), it leads to the endergonic formation of intermediate **I2′** with a Gibbs energy of −1.52 eV and an overall charge +1. Conversely, if the proton is transferred from LuHOTf (pathway B), it results in the exergonic formation of intermediate **I2** with a Gibbs energy of −2.52 eV and an overall charge of +2. The significant energy difference between intermediates **I2′** and **I2** (i.e., 1.00 eV) makes pathway B energetically more favorable.

In subsequent steps, **I2** and **I2′** each undergo another PCET step to form **I3** and **I3′**, respectively. This H atom transfer is significantly exergonic, rendering the process irreversible and leading to a divergence in pathways. Notably, intermediate **I3**, resulting from the external proton transfer (pathway B), exhibits an interesting structure characterized by a 3-membered {Fe–N–O} metallocycle, resembling the side-on coordination of TEMPO, which is isoelectronic to H_2_NO radical, previously observed to nickel.^90^ Conversely, intermediate **I3′**, obtained via intramolecular proton transfer and outer-sphere electron transfer (pathway A), maintains a linear configuration with an NH_2_O radical bound to Fe through the N atom.

While **I3** is considerably more stable than **I3′** by −1.50 eV, the stability of these species relative to their respective structural conformers varies. Specifically, the linear **I3′** with two deprotonated ligand arms (pathway A) is more stable by −0.30 eV compared to its cyclic counterpart **I3′a**. Conversely, the cyclic intermediate **I3** (pathway B) with fully protonated ligand arms is less stable by 0.07 eV than its linear analogue, denoted as **I3a**.

**Figure 3:**
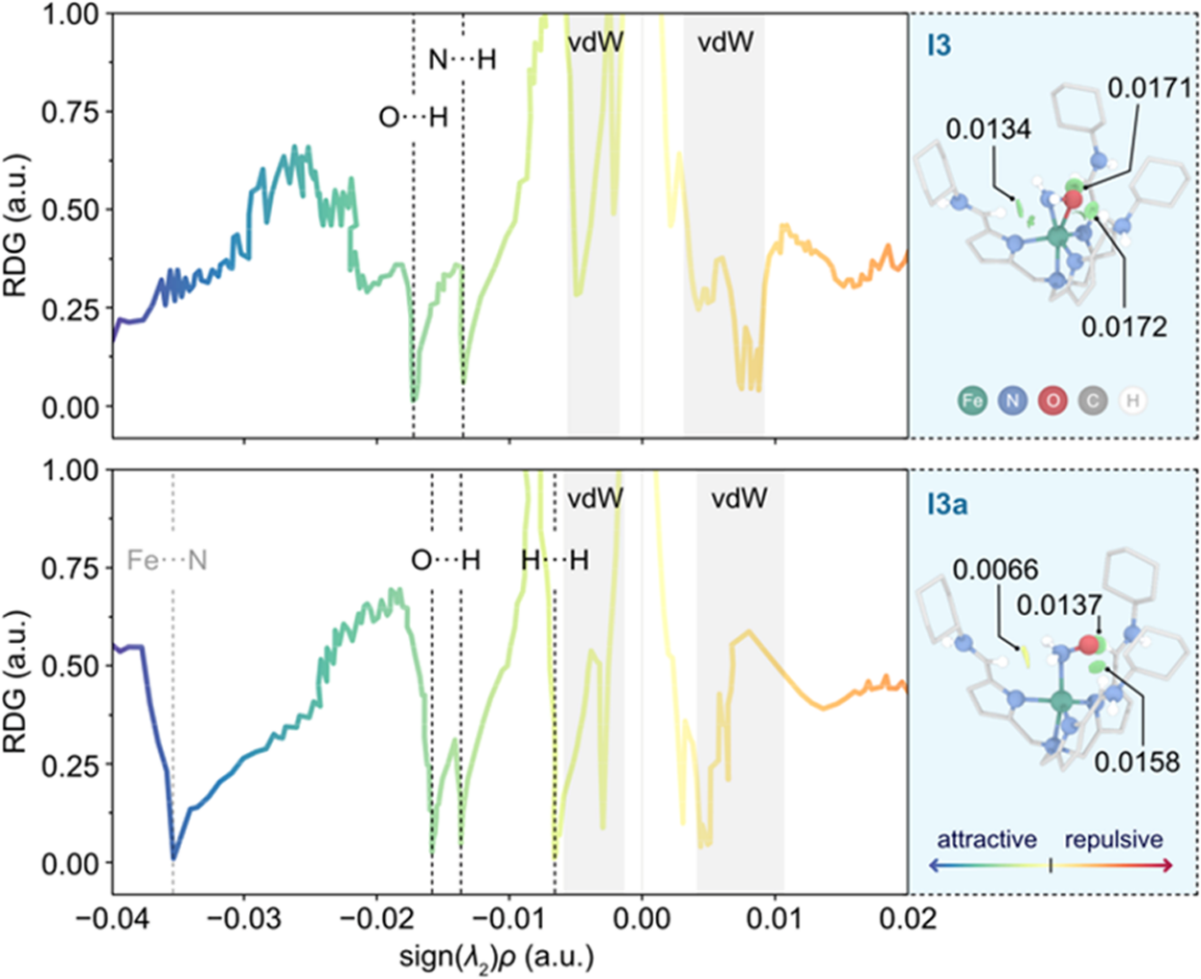
NCI analysis of complexes **I3** (top) and **I3a** (bottom), showing the reduced density gradient (RDG) plotted against the product of the sign of the second eigenvalue of the Hessian matrix, sign(λ_2_)ρ, and the electron density, (ρ). Additional details on this analysis are provided in the SI. In the NCI plot, attractive (repulsive) interactions are indicated by blue (red) shades, with darker (lighter) shades denoting stronger (weaker) interactions. The inset highlights the most relevant attractive NCIs as isosurfaces (isovalue = 0.6 au), along with their associated ρ values (in a.u.).

To better understand the origin of the energy difference between **I3** and **I3a**, formed along the most favorable pathway B, we performed noncovalent interaction (NCI) analysis. This analysis uses electron density distributions to provide semiquantitative insights into both the nature and strength of NCIs between defined fragments in a molecule (see SI for further details). Specifically, we aimed to investigate the interactions between the secondary coordination sphere of the ligand and the bound NH_2_O radical. For this purpose, the molecules were partitioned into two fragments: the {NH_2_O} moiety and the {FeL} component (where L = N(afa^Cy^)_3_). The generated NCI plot and the corresponding three-dimensional isosurfaces representing the NCIs are illustrated in Figure 3.

The O···H electrostatic interactions are notably stronger in the 3-membered {Fe–N–O} metallocycle **I3** (shifted to more negative values of sign(λ_2_)ρ) with ρ values of 0.0171 and 0.0172 au, compared to those in linear {Fe–N–O} **I3a**, which are 0.0137 and 0.0158 au This enhanced stabilization in **I3** arises from the favorable orientation of the NH_2_O group within the cyclic structure, optimally aligning the oxygen’s lone pairs for effective electrostatic interactions with the C–H moieties of the ligand. Additionally, **I3** exhibits a relatively weak electrostatic interaction between the N and H atoms, characterized by a ρ = 0.0137 au In contrast, the corresponding H···H interactions in **I3a** are even weaker (ρ = 0.0066 au), typical for van der Waals interactions.^91^ Altogether, the NCI interactions depicted in the inset of Figure 3 illustrate the greater stabilization in **I3** compared to **I3a**.

While the NCI analysis provides valuable insight, it does not fully account for the higher stability of the linear intermediate **I3′** relative to its cyclic counterpart **I3′a**. We attribute this apparent discrepancy to the instability of the NH_2_O radical in the cyclic intermediate **I3′a**, likely due to increased electron donation from the two deprotonated ligand arms. In contrast, this electronic repulsion is minimized in the linear analogue **I3′** by increasing the radical character on the O atom of the {NH_2_O} moiety, away from the deprotonated ligand arms. This is further supported by the presence of a weak Fe–N bond in **I3a** with significant noncovalent character in the region below −0.030 au (Figure 3), a feature not presented in **I3**.

The subsequent PCET process from **I3** leads to the formation of the NH_2_OH-bound intermediate **I4** (Figure 2), consistent with our independent NH_2_OH reduction experiments. Calculations show that the N–Fe bound isomer (**I4**) is favored over the O–Fe counterpart (**I4a**, Scheme S2a) by −0.28 eV. Considering the stoichiometry of the external acid added (3 equiv), which is fully consumed in the prior reaction steps through the pathway B, sequential PCET steps will source the protons from the ligand. Progressive intramolecular proton transfer and outer-sphere electron transfer to **I4** lead to the release of a water molecule, forming **I5**, a species featuring an NH_2_ radical bound to a high-spin Fe(II) center. The analogous structure with an NH_2_ anion attached to a low-spin Fe(III) center was also computed (**I 5s**, Scheme S2b), but it is higher in energy by +0.18 eV, suggesting a stabilization preference for the Fe(II) oxidation state and the nitrogen-based radical.

The next PCET step from **I5** is highly exergonic by −2.60 eV, resulting in the formation of **I6** with the generated NH_3_ still bound to the Fe(II) center. From **I6**, the eventual release of NH_3_ occurs (**I7**), followed by the binding of a water molecule, which transfers one of its protons to the ligand arm, generating complex **4**, as evidenced by ^1^H NMR spectroscopy.

For pathway A, calculations revealed an intriguing switch in binding preferences, where the O–Fe bound isomer (**I4**′) is favored over the N–Fe variant by −0.33 eV (**I4**′**a**, Scheme S3). This reversal in affinity leads first to the elimination of NH_3_, while the OH group remains bound to Fe, yielding the intermediate **I6′**. A series of PCET steps then follow, and ultimately, both pathways A and B converge to form the same final products: **4** and NH_3_.

Overall, this computational study emphasizes the significant influence of the ligand’s secondary coordination sphere and the preferential sourcing of protons from the external acid LuHOTf (pathway B) in these two extreme scenarios. While the PCET is likely extremely complicated, these insights will likely inform the design and optimization of analogous systems in future developments.

## Conclusions

This work demonstrates a selective, stepwise reduction of nitrate or nitrite to either dinitrogen or ammonia, with the outcome controlled by the choice of sacrificial reductant used in the nitric oxide reduction step.^15,78^ While complex **1** is incredibly successful in deoxygenation reactions, the addition of external reagents in the crucial nitric oxide reduction step allows for novel reactivity in this system, such as N–N coupling to form nitrous oxide. In the first pathway, selective nitrite reduction to dinitrogen was achieved without requiring strong reductants. When using PPh_3_ as a sacrificial reductant in the nitric oxide reduction step, quantitative O=PPh_3_ production was observed, with nitrous oxide as the sole N-containing product. In the second step, the oxidation of complex **1** by nitrous oxide yielded an iron-oxo complex (**3**) alongside dinitrogen, closely mimicking the N-containing intermediates in the biological denitrification processes.

In the nitrate-to-ammonia pathway, strong reductants are required to reduce the iron nitrosyl species **2**, formed in the reduction of nitrate or nitrite by **1**, leading to relatively low yields of ammonia (28.7%). The electronic structure and iron oxidation state of complex **2** were examined through CASSCF and CASCI calculations, identifying the species as primarily Fe(III)(*S* = 3/2)–NO^–^(*S* = 1), in agreement with previous reported EPR spectroscopy.^15^

Analogous to ccNiR-mediated nitrate reduction, our results suggest a hydroxylamine intermediate in the ammonia production pathway, as supported by complex **1**’s ability to directly reduce hydroxylamine to ammonia. This hypothesis is further reinforced by computational studies, which identify a bound hydroxylamine intermediate along the lowest-energy pathway in the reduction of **2**. Additionally, NCI analysis highlights the crucial role of the ligand’s secondary coordination sphere in stabilizing an N-bound hydroxylamine radical, favoring it over a cyclic intermediate. This system is a rare example of a molecular catalyst selectively producing either ammonia or dinitrogen from nitrate or nitrite, with product selectivity governed by the choice of reductant at the crucial NO reduction step. These findings provide valuable insights for the rational design of related catalytic systems, showcasing methods to overcome difficult reaction steps and paving the way for future advancements in selective nitrate reduction.

## Data Availability

All the DFT data underlying this work, including the Cartesian coordinates and energies of all the modelled structures, is free and openly accessible via the following ioChem-BD online dataset: DOI: 10.19061/iochem-bd-6-402.

## Supporting Information Available

Experimental, spectral, and computational details (PDF).
