# Supplementary material for: Selective Stepwise Reduction of Nitrate and Nitrite to Dinitrogen or Ammonia
Source: J Am Chem Soc. 2025 Feb 28;147(10):8444–54. doi: 10.1021/jacs.4c16585 (PMC11912340; doi:10.1021/jacs.4c16585)
Supplement: Supplementary file 1 — ja4c16585_si_001.pdf [file ja4c16585_si_001.pdf]

Selective Stepwise Reduction of Nitrate and Nitrite to Dinitrogen or Ammonia

Jewelianna M. Moore,[a] Tabitha J. Miller,[b] Manting Mu,[c] Marconi N. Peñas-Defrutos,[c,d] Kelly L. Gullett,[b] Lindsey S. Elford,[a] Sebastian Quintero,[a] Max García-Melchor,*[c,e,f] and Alison R. Fout*[a]

[a] J. M. Moore, L. S. Elford, S. Quintero, Prof. A. R. Fout
Department of Chemistry
Texas A&M University
580 Ross St. College Station, TX 77843, USA.
E-mail: fout@tamu.edu

[b] Dr. T. J. Miller, Dr. K. L. Gullett
School of Chemical Sciences
University of Illinois at Urbana-Champaign
600 S. Matthews Ave. Urbana, IL 61801, USA

[c] M. Mu, Dr. M. N. Peñas-Defrutos, Prof. M. García-Melchor
School of Chemistry, CRANN and AMBER Research Centres
Trinity College Dublin, College Green, Dublin 2 (Ireland)
E-mail: garciamm@tcd.ie

[d] Dr. M. N. Peñas-Defrutos
IU CINQUIMA, Química Inorgánica, Facultad de Ciencias,
Universidad de Valladolid, 47071 Valladolid (Spain)

[e] Prof. M. García-Melchor
Center for Cooperative Research on Alternative Energy (CIC EnergiGUNE),
Basque Research and Technology Alliance (BRTA), Alava Technology Park,
Albert Einstein 48, 01510 Vitoria-Gasteiz (Spain)

[f] Prof. M. García-Melchor
Ikerbasque, Basque Foundation for Science,
Plaza de Euskadi 5, 48009 Bilbao (Spain)

Table of Contents

General Considerations……S2
General Procedure for GC Head Space Analysis……S2
Reduction of Complexes to form Ammonia……S2
Synthesis of ^{15}**N-2**……S3
Method of Quantification of Ammonia……S4
Quantification of Ammonia……S4
Reduction of Nitrite to Nitrous Oxide……S5
Oxidation of **1** by Nitrous Oxide……S5
Spectra for the Reduction of Nitrite to Ammonia or Nitrogen……S6
Crystallographic Structure……S15
Crystallographic Parameters……S16
Computational Section……S17

General Considerations. Unless otherwise stated, all manipulations were carried out in an MBraun inert atmosphere drybox under an atmosphere of nitrogen or using standard Schlenk techniques. Solvents for air- and moisture-sensitive manipulations were dried and deoxygenated using a Glass Contour System (SG Water USA, Nashua, NH) following literature procedure and stored over 4Å molecular sieves (3Å for MeCN) purchased from Strem prior to use. Celite 545 (J. T. Baker) was heated to 150°C under dynamic vacuum for 24h prior to use in the drybox. All reagents were purchased from commercial sources and used as received unless otherwise noted. $[(n\text{-Bu})_4N]NO_2$ ($TBANO_2$) was recrystallized from tetrahydrofuran and hexanes under an inert atmosphere prior to use. $Na^{15}NO_2$ was purchased from Cambridge Isotope Laboratories and used as received. $Fe(OTf)_2{\cdot}2MeCN$ was prepared according to literature procedure.[1] KC_8 was prepared by heating potassium metal with graphite in a 1:8 ratio at 80° C for 4 hours. ***Caution!*** Potassium metal and its intercalation compound (KC_8) are both pyrophoric and should be handled with extra care; both the synthesis and use of the reagent were carried out under a nitrogen atmosphere. $[N(afa^{Cy})_3FeNO](OTf)_2$,[2] $H_3[N(pi^{Cy})_3]$,[3] $N(afa^{Cy})_3Fe(OTf)_2$,[3] and 2,6-lutidinium triflate (LuHOTf)[4] were synthesized according to literature procedure. NMR solvents (CD_2Cl_2, $CDCl_3$, acetonitrile-d_3, and DMSO-d_6) were purchased from Cambridge Isotope Laboratories, degassed, and stored over 4Å molecular sieves prior to use. NMR spectra were recorded at ambient temperature on a Varian spectrometer operating at 500 MHz (^1H NMR) and referenced to the peak of the residual solvent (δ in parts per million and J in Hz). Solid-state infrared spectra were measured using a PerkinElmer Frontier FT-IR spectrophotometer equipped with a KRS5 thallium bromide/iodide universal attenuated total reflectance accessory.

General Procedure for GC Head Space Analysis: Reactions were carried out in an argon drybox unless otherwise stated. A 4 mL scintillation vial was charged with compounds of choice and a 3 mm by 8 mm stir bar with a box atmosphere below 1.0 ppm oxygen. Acetonitrile (1.5 mL) was added, and the vial was quickly capped with an 8 mm septa and taped with electrical tape. Reactions were stirred in the drybox for 16 hours and brought out of the box for analysis. 0.3 mL of the head space was inserted into the Agilent Trace 1300 gas chromatograph. The GC was attached with a thermal conductivity detector and a custom-made 120 cm stainless steel column packed with Carbosieve-II was used to identify gases. The column was kept at a temperature of 200 °C and argon was used to carry the gas during the separation. The detector was set to a temperature of 250 °C.

Direct Reduction of NO_3^- to NH_3. A 20 mL scintillation vial was charged with **1** (112 mg, 0.12 mmol, 1 equiv.), $TBANO_3$ (12 mg, 0.04 mmol, 0.33 equiv.), and 4 mL of THF. The resulting mixture was stirred for 16 hours after which KC_8 (81 mg, 0.6 mmol, 5 equiv.) and LuHOTf (93 mg, 0.36 mmol, 3 equiv.) were added along with an HCl trap (2 mL of 0.2 M $HCl{\bullet}Et_2O$ in a 4 mL scintillation vial). The 20 mL reaction vial was quickly capped and taped. After stirring for 6 hours, the HCl trap was removed and dried via vacuum. ^1H NMR analysis of the HCl trap solids displayed of NH_4Cl, confirming production of ammonia.

Control Reduction of NO(g) using KC_8. A 50 mL Schlenk round bottom flask was charged with KC_8 (34 mg, 0.25 mmol) suspended in 5 mL of THF under inert atmosphere. NO(g) was vacuum transferred to the vessel. The reaction was stirred overnight and quenched and provided protons with 0.1 M HCl (2.5 mL, 0.25 mmol). Analysis of the quenched reaction via ^1H NMR spectroscopy showed no formation of NH_4Cl.

Reduction of $[N(afa^{Cy})_3Fe(NO)](OTf)_2$ using KC_8. A 20 mL scintillation vial was charged with **2** (38.6 mg, 0.04 mmol), KC_8 (27.0 mg, 0.2 mmol, 5 equiv), and 2,6-lutidinium triflate (30.9 mg, 0.12 mmol, 3 equiv). THF (3 mL) was added, and the resulting mixture was stirred for 1 hour before filtering to remove graphite. The resulting red solution was then dried in vacuo. The red product was extracted using dichloromethane (2 x 4 mL) and was then dried in vacuo. The product was identified as previously characterized complex $N(afa^{Cy})_2(pi^{Cy})FeOH$ (**4**)[3] by ^1H NMR spectroscopy.

Reduction of $[N(afa^{Cy})_3FeNO](OTf)_2$ using NaK. A 20 mL scintillation vial was charged with cleanly cut pieces of Na (2.9 mg, 0.125 mmol, 2.5 equiv) and K (4.9 mg, 0.125 mmol, 2.5 equiv). Using a clean plastic spatula to mix, a liquid alloy was formed. To this was added **2** (48.3 mg, 0.05 mmol) and 2,6-lutidinium triflate (38.6 mg, 0.15 mmol, 3 equiv), as well as a glass stir bar and THF (4 mL). After stirring overnight, the mixture had become a red-brown solution. Volatiles were removed under vacuum. The red product was extracted from the resulting residue using DCM (2 x 5 mL) and filtering through a pad of Celite. The combined DCM filtrate was dried under vacuum to yield a red-brown powder. The product was identified as **4** by ^1H NMR spectroscopy. Recrystallization yield: 18.5 mg (56.7 %).

Synthesis of $[N(afa^{Cy})_3Fe(^{15}NO)](OTf)_2$. A 20 mL scintillation vial was charged with $N(afa^{Cy})_3Fe(OTf)_2$ (**1**) (93.6 mg, 0.1 mmol) in 5 mL THF. While stirring, $Na^{15}NO_2$ (7.0 mg, 0.1 mmol) was weighed by difference and added as a solid. The mixture was stirred for 1 hour, and then dried under vacuum. To the residue was added a 3:1 THF/Et_2O solution (4 mL). The resulting mixture was filtered through a filter pipet to remove the insoluble byproduct **3**.[2] The filtrate was dried under vacuum to yield a brown powder. The ^1H NMR spectrum in MeCN-d_3 matched that for the published spectrum of unlabeled complex.[2,5] IR: 1717 cm^{-1} (N=O).

Reduction of NH_2OH by $N(afa^{Cy})_3Fe(OTf)_2$. A 20 mL scintillation vial was charged with $(NH_2OH)_2{\cdot}H_2SO_4$ (4.1 mg, 0.025 mmol) or $NH_2OH{\cdot}HCl$ (3.5 mg, 0.05 mmol) and **1** (93.6 mg, 0.1 mmol) in 3 mL THF. To this suspension was added approx. 4 equiv NEt_3 (40 mg, 0.4 mmol). After stirring for 18 hours, the reaction was dried under vacuum, yielding a dark brown residue. The dark brown product was isolated by extraction using DCM (2 x 4 mL) and was then dried under vacuum to yield a dark brown powder. The product was identified as **3** by ^1H NMR spectroscopy.[2] Recrystallization yield: 56.9 mg, (70.6 %).

General Procedure for Detection of NH_3 via ^1H NMR Spectroscopy. A 30 mL jar was charged with compound of choice and the appropriate amount of KC_8. A 4 mL vial was charged with 2 mL 1.0 M $HCl{\cdot}Et_2O$ and placed inside the reaction jar. If LuHOTf was used, it was added to the THF (4 mL) before addition to the jar. Otherwise, THF (4 mL) was added directly to the jar. Upon addition of solvent, the jar was immediately capped and taped shut. After stirring for 4 hours, the inner vial was removed, and the contents dried under vacuum. A ^1H NMR spectrum of the acid trap was obtained in DMSO-d_6.

Buffered Indophenol Method. Quantification of NH_3 was done by a modified indophenol method.[6] A buffered catalyst solution was prepared from sodium phosphate tribasic (0.3 g, 0.79 mmol), sodium citrate tribasic (0.3 g, 1.0 mmol), Na_2EDTA (0.03 g, 0.081 mmol), and phenol (0.6 g, 6.4 mmol). After dissolving to approximately 8 mL in DI H_2O, sodium nitroprusside (0.002 g, 0.008 mmol) was added, and the solution diluted to 10.0 mL. To avoid catalyst decomposition, the solution was stored in either an amber bottle or a taped vial. An alkaline solution was prepared from sodium hydroxide (0.4 g, 10 mmol) and commercial bleach (0.157 mL) diluted to 10.0 mL. Stock solutions of NH_4Cl were used to prepare a calibration curve (Figure S1). UV-Vis samples were prepared by mixing 2 mL of a reaction sample with 0.8 mL of the catalyst solution and 1.2 mL of the alkaline solution. Samples were left to develop for 45 minutes before measuring their absorbance at 635 nm.

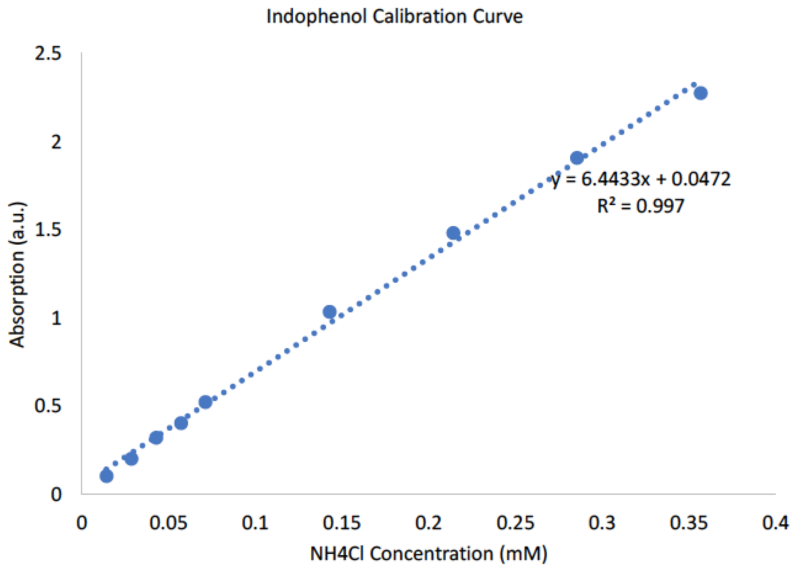

Figure S1. Calibration curve for the calorimetric assay used to quantify ammonia production.

General Procedure for Ammonia Quantification. A 50 mL storage vessel was charged with the appropriate amount of KC_8 or NaK, LuHOTf, and compound of choice. THF (4 mL) was quickly added, followed by sealing the vessel with a Teflon cap. The reaction was then allowed to stir for 1 hour outside of the glove box (overnight when NaK is used). For the quantification of NH_3, reaction volatiles were collected by vacuum transfer into concentrated HCl (aq). After warming to room temperature while stirring, volatiles were removed from the HCl trap. To the resulting residue, 2 mL of DI H_2O (10 mL for NaK reactions) was added to extract NH_4Cl, and these samples were further diluted to achieve absorbance values within the range of the calibration curve.

Ammonia Yields:

Reduction of [N(afa^{Cy})$_3$Fe(NO)](OTf)$_2$ by KC_8: KC_8 (33.8 mg, 0.25 mmol); **2** (48.3 mg, 0.05 mmol); LuHOTf (38.6 mg, 0.15 mmol). Yield of Ammonia: 0.0047 mmol (9.5 %).

Reduction of [N(afa^{Cy})$_3$Fe(NO)](OTf)$_2$ by NaK: Na (2.9 mg, 0.125 mmol); K (4.9 mg, 0.125 mmol); **2** (48.3 mg, 0.05 mmol); LuHOTf (38.6 mg, 0.15 mmol). Yield: 0.014 mmol (28.7 %).

Control Reduction of N(afa^{Cy})$_3$Fe(OTf)$_2$ by NaK: Na (2.9 mg, 0.125 mmol); K (4.9 mg, 0.125 mmol); **2** (46.8 mg, 0.05 mmol); LuHOTf (38.6 mg, 0.15 mmol). Yield: 0.0009 mmol (1.8 %).

Control Reduction of N(pi^{Cy})$_3$ by NaK: Na (2.9 mg, 0.125 mmol); K (4.9 mg, 0.125 mmol); N(pi^{Cy})$_3$ (29.1 mg, 0.05 mmol); LuHOTf (38.6 mg, 0.15 mmol). Yield: 0.0020 mmol (4.1 %).

Reduction of NH_2OH by N(afa^{Cy})$_3$Fe(OTf)$_2$: 1 (46.8 mg, 0.05 mmol); $NH_2OH{\cdot}HCl$ (3.5 mg, 0.05 mmol); KOH (2.8 mg, 0.05 mmol). Yield: 0.015 mmol (29.8 %).

Reduction of NO_2^- to N_2O: A 4 mL scintillation vial was charged with **1** (28 mg, 0.03 mmol), $TBANO_2$ (8.6 mg, 0.03 mmol), PPh_3 (7.9 mg, 0.03 mmol), NEt_3 (approx. 5 mg, 0.05 mmol), and a 3mm by 8 mm stir bar with a box atmosphere below 1.0 ppm oxygen. Acetonitrile (1.5 mL) was added, and the vial was quickly capped with an 8 mm septa and taped with electrical tape. The resulting mixture was stirred for 16 hours. A 0.3 mL aliquot was taken of the head space by gas tight Hamilton syringe. The formation of nitrous oxide was confirmed by gas chromatography head space analysis. The resulting brown solution was dried in vacuo. The solid was extracted first with ether (6 x 1.5 mL) to remove phosphine products. The formation of $O{=}PPh_3$ was confirmed by ^{31}P NMR spectroscopy (quantitative yield by integration with $S{=}PPh_3$ (8.8 mg, 0.015 mmol)). The metal product was extracted by acetonitrile and was determined to be **3** by ^1H NMR spectroscopy.

Oxidation of $N(afa^{Cy})_3Fe(OTf)_2$ by N_2O: A dry 25 mL Schlenk tube was charged with a stir bar, **1**, and 3 mL of acetonitrile containing NEt_3 (approx. 5 mg, 0.05 mmol). The Schlenk tube was brought out of the drybox and was evacuated using Schlenk techniques. The solution was frozen using a dry ice acetone bath and was exposed to N_2O (1 atm in Schlenk line). The vessel was sealed and brought into the dry box after coming to room temperature to stir for four hours. The head space and solvent were removed in vacuo. **3** was confirmed to be the major product by ^1H NMR spectroscopy.

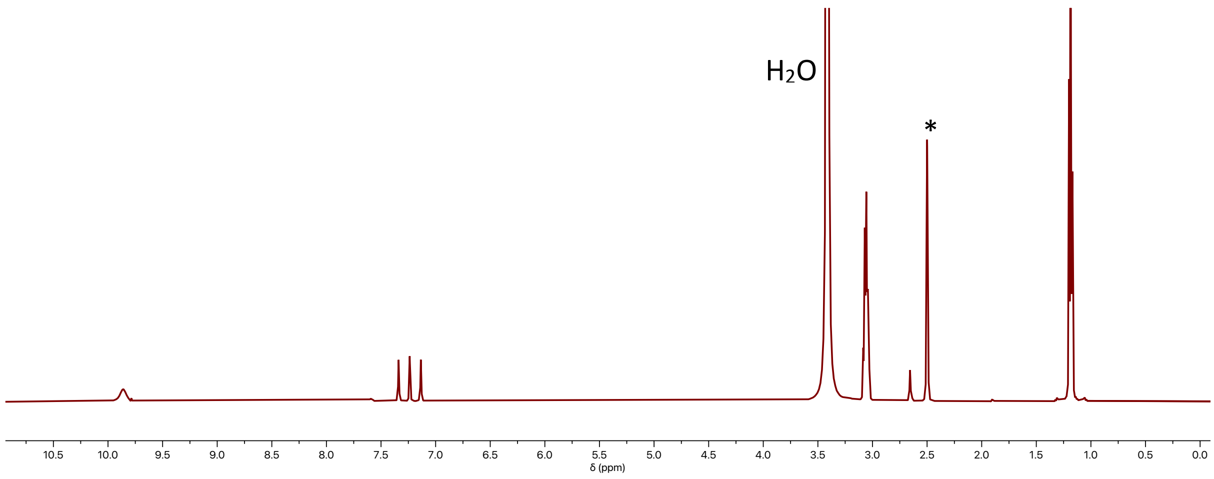

Figure S2. ^{1}H NMR Spectrum the HCl trap in the reduction of nitrate to ammonia (DMSO-d_6).

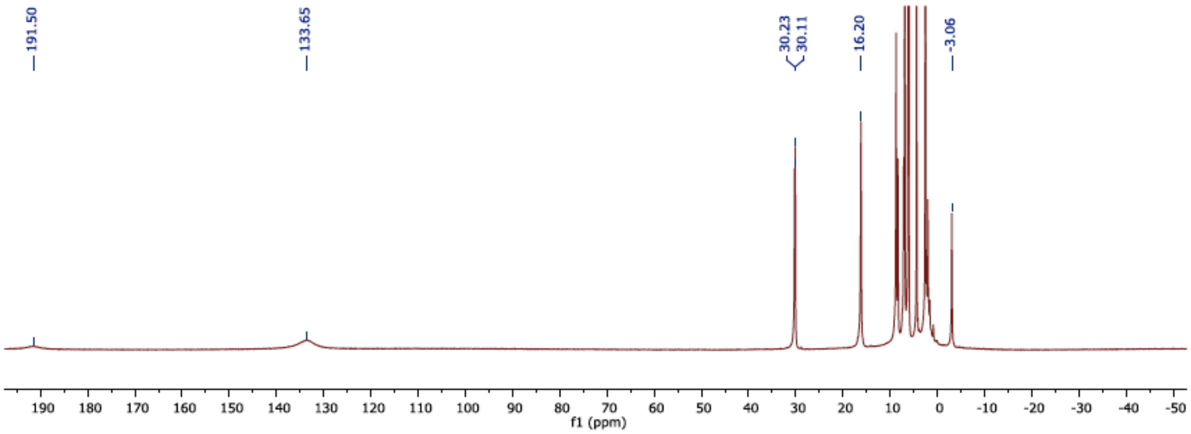

Figure S3. ^{1}H NMR Spectrum of isolated product **4** from reduction of **2** with KC_8 (acetonitrile-d_3).

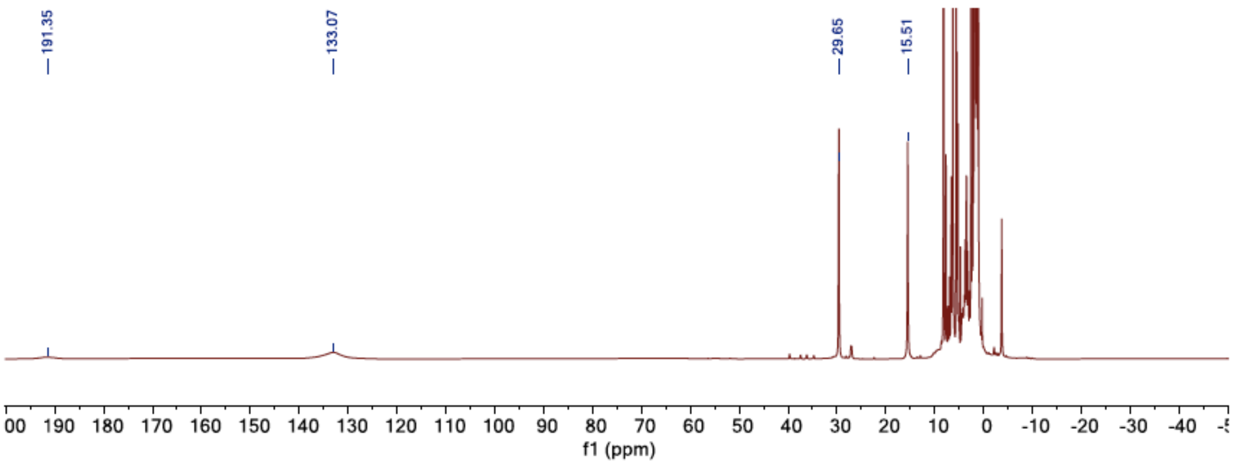

Figure S4. ^{1}H NMR Spectrum of isolated product **4** from reduction of **2** with NaK (dichloromethane-d_2).

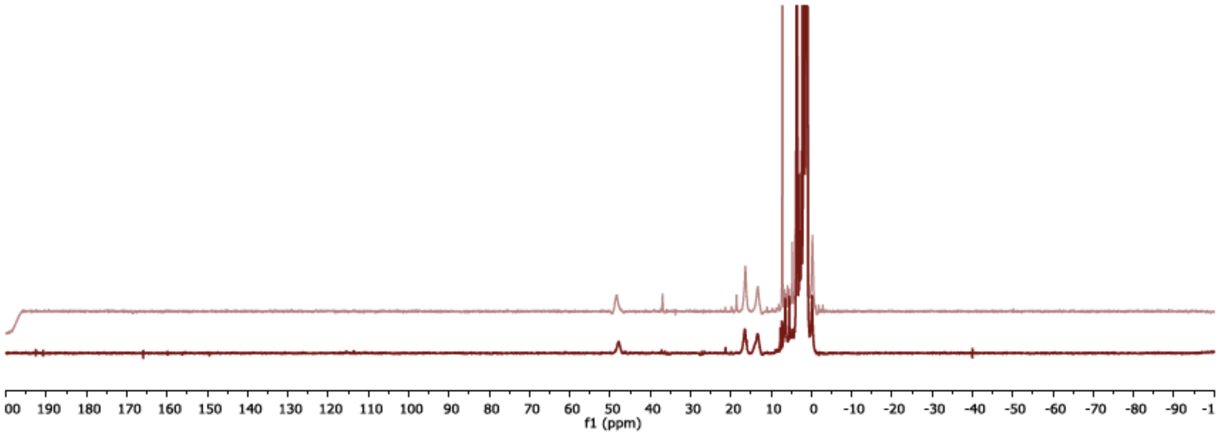

Figure S5. ^{1}H NMR Spectra of **2** (top) and **^{15}N-2** (bottom) (acetonitrile-d_3).

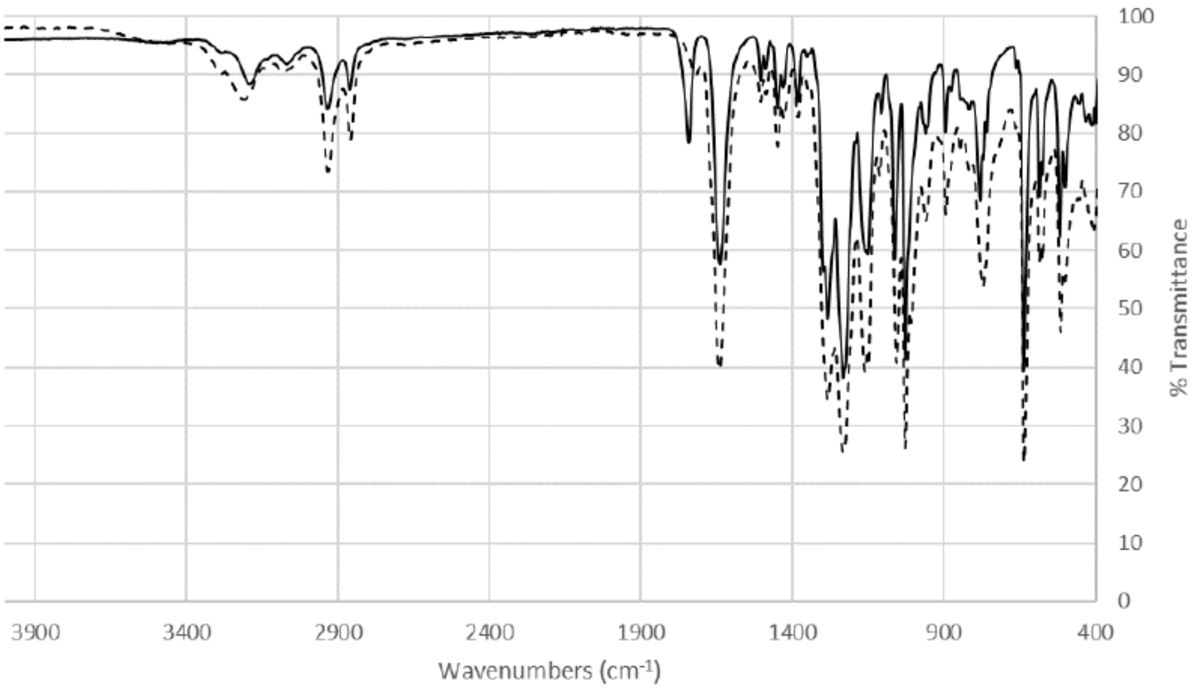

Figure S6. FTIR-ATR spectra of **2** (solid line) and **^{15}N-2** (dashed line).

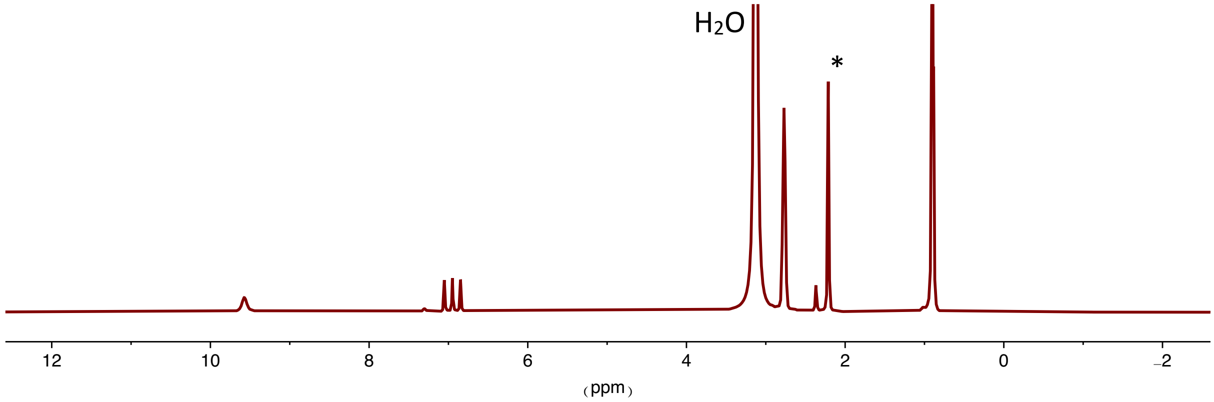

Figure S7. ^{1}H NMR spectra of the HCl trap of the reduction of **^{1}2** by 5 equiv KC_8 and 3 equiv LuHOTf displaying formation of $^{14}NH_4Cl$ (6.95 ppm, t, 51 Hz) (DMSO-d_6).

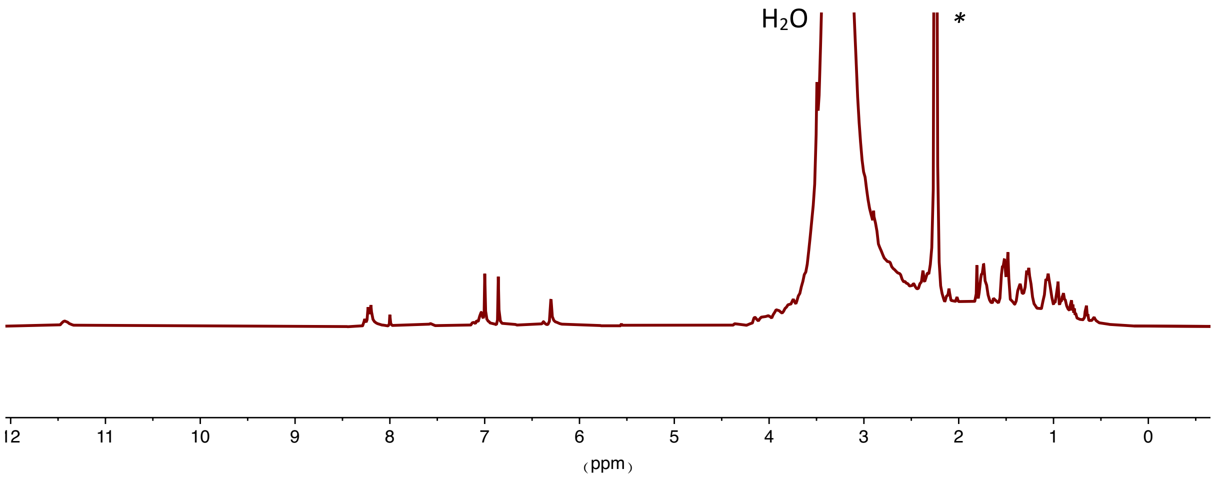

Figure S8. ^{1}H NMR spectra of the HCl trap of the reduction of **^{15}N-2** by 5 equiv KC_8 and 3 equiv LuHOTf displaying formation of $^{15}NH_4Cl$ (6.95 ppm, d, 71 Hz) (DMSO-d_6).

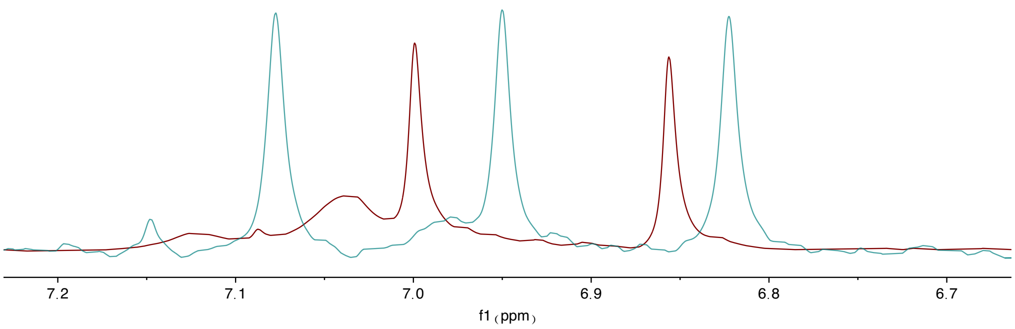

Figure S9. Selected region of ^{1}H NMR spectra of the HCl trap of the reduction of **2** (blue) or **^{15}N-2** (red) by 5 equiv KC_8 and 3 equiv LuHOTf displaying formation of NH_4Cl (6.95 ppm, t, 51 Hz) or $^{15}NH_4Cl$ (6.95 ppm, d, 71 Hz) (DMSO-d_6).

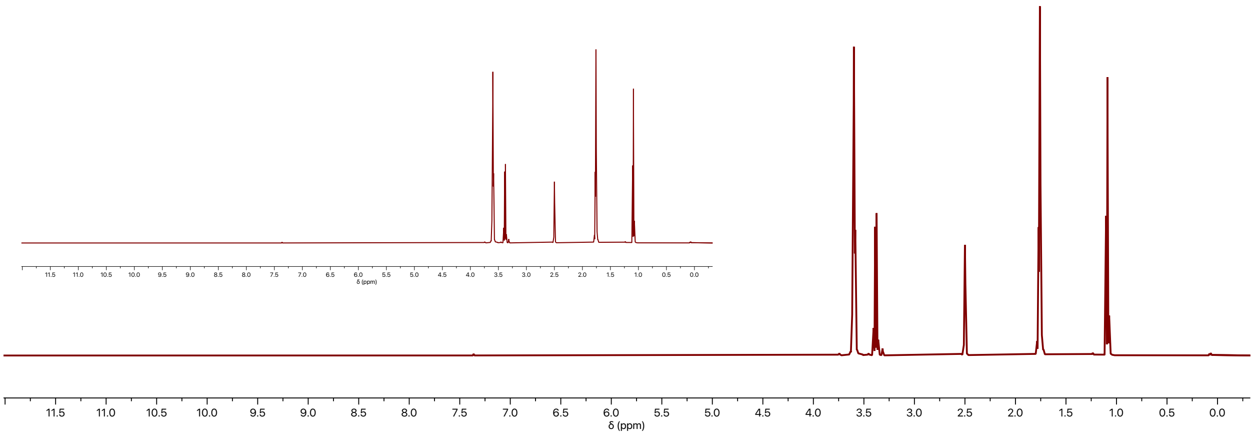

Figure S10. ^{1}H NMR spectrum of the control reduction of pure NO gas with inlay of region of interest, showing no production of NH_4Cl (DMSO-d_6).

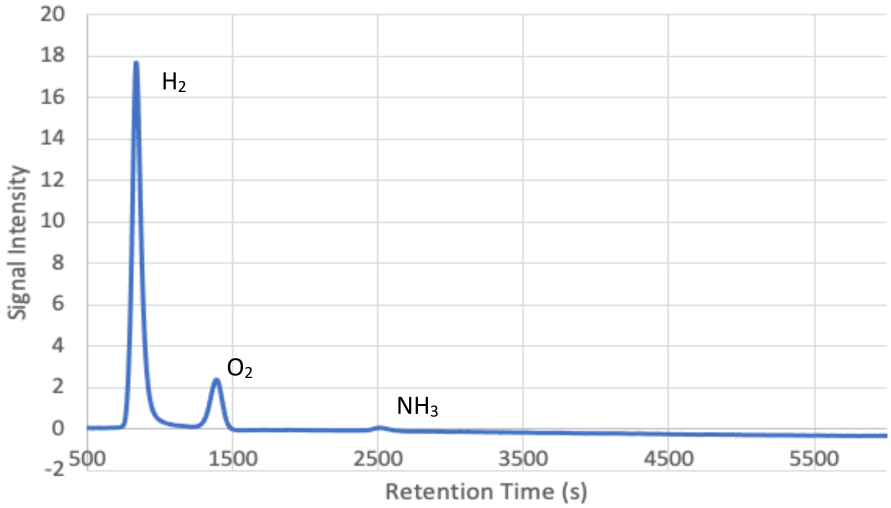

Figure S11. Gas chromatography headspace analysis of the reduction of **2** by 5 equiv KC_8, showing formation of ammonia as the sole nitrogen containing product. The presence of O_2 is due to atmospheric contamination during injection.

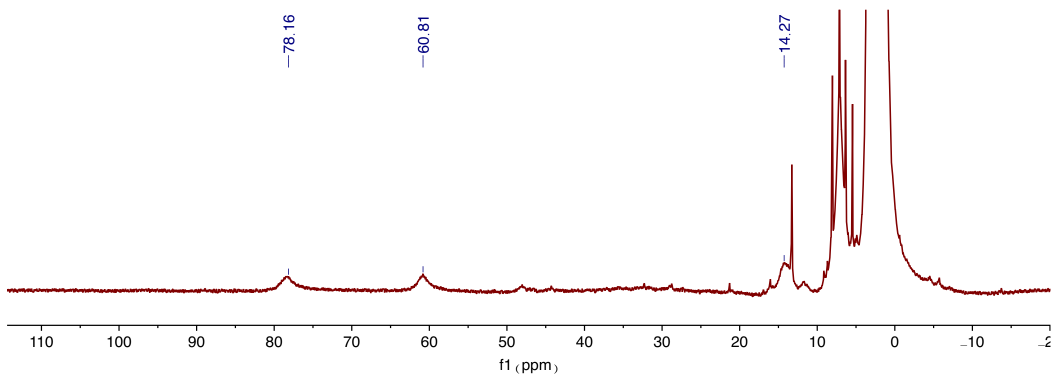

Figure S12. ^1H NMR spectrum of product **3** from reduction of $NH_2OH•HCl$ with **1** and 4 equiv NEt_3 (acetonitrile-d_3).

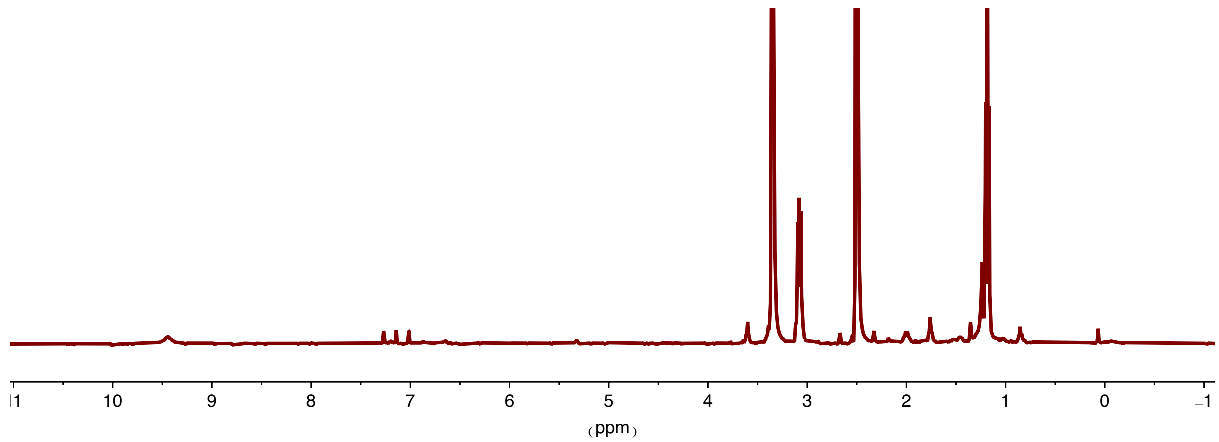

Figure S13. Full ^1H NMR spectrum the HCl trap of the reduction of $NH_2OH•HCl$ by **1** with 4 equiv of NEt_3 displaying the formation of NH_4Cl (DMSO-d_6).

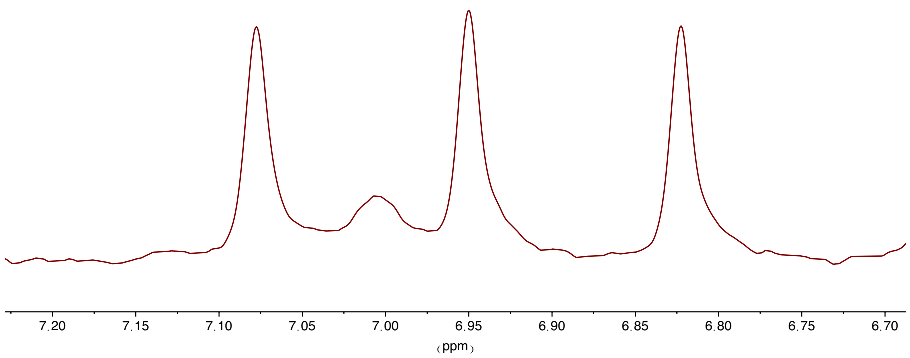

Figure S14. Selected Region of ^1H NMR spectrum the HCl trap of the reduction of NH_2OH•HCl by **1** with 4 equiv. of NEt_3 displaying the formation of NH_4Cl (DMSO-d_6).

Spectra for the reduction of nitrite to nitrogen

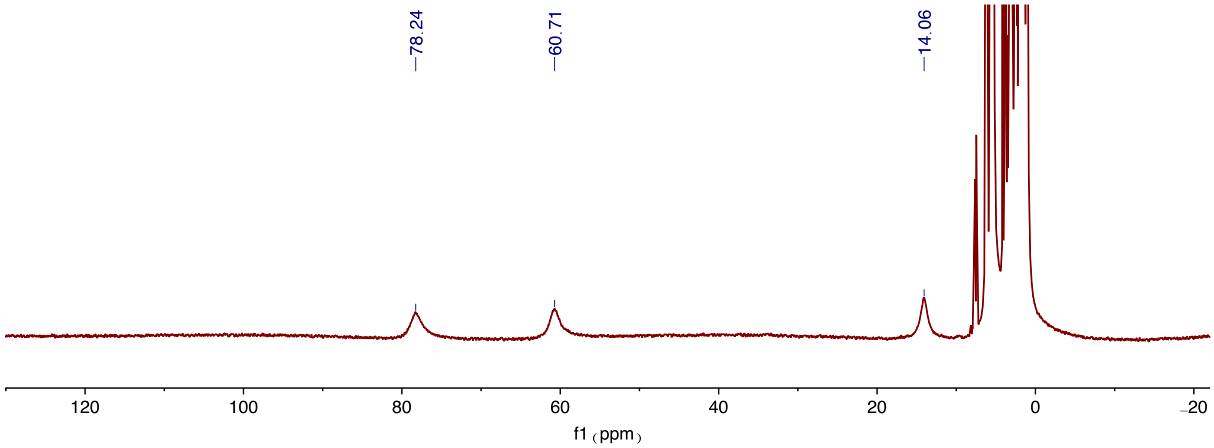

Figure S15. ^{1}H NMR spectrum of product **3** from the reduction of tetrabutylammonium nitrite by 1 equiv **1** and 1 equiv PPh_3 (acetonitrile-d_3).

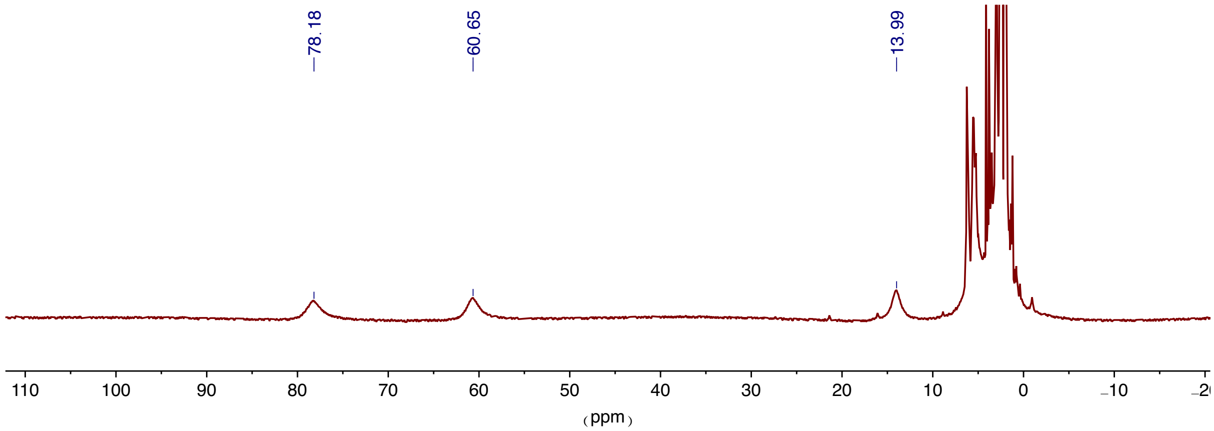

Figure S16. ^{1}H NMR spectrum of product **3** from the reaction of **1** and nitrous oxide (acetonitrile-d_3).

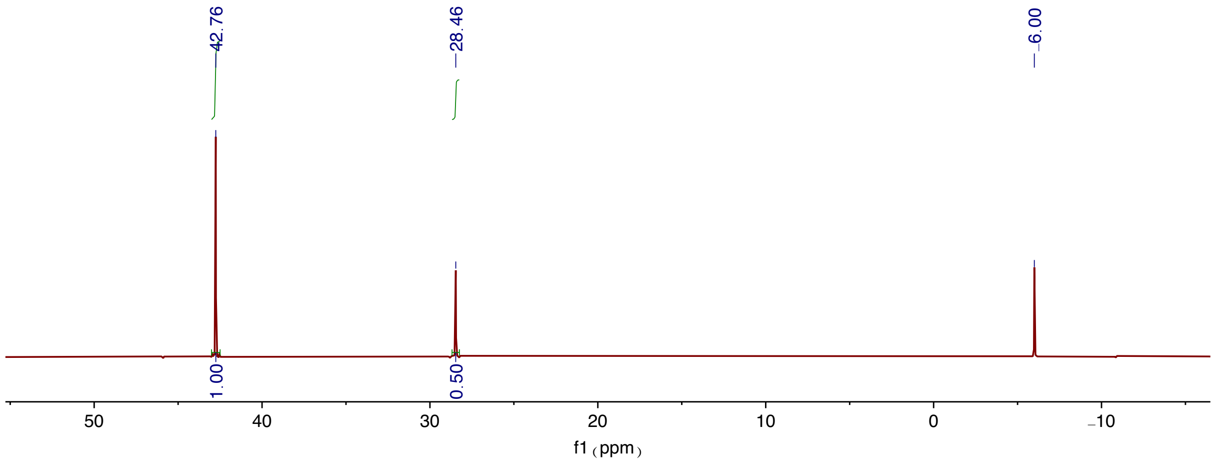

Figure S17. ^{31}P NMR spectrum of product from the reduction of tetrabutylammonium nitrite by 1 equiv **1** and 1 equiv PPh_3 (Chloroform-d_1). $S=PPh_3$ was added as an internal reference for integration.

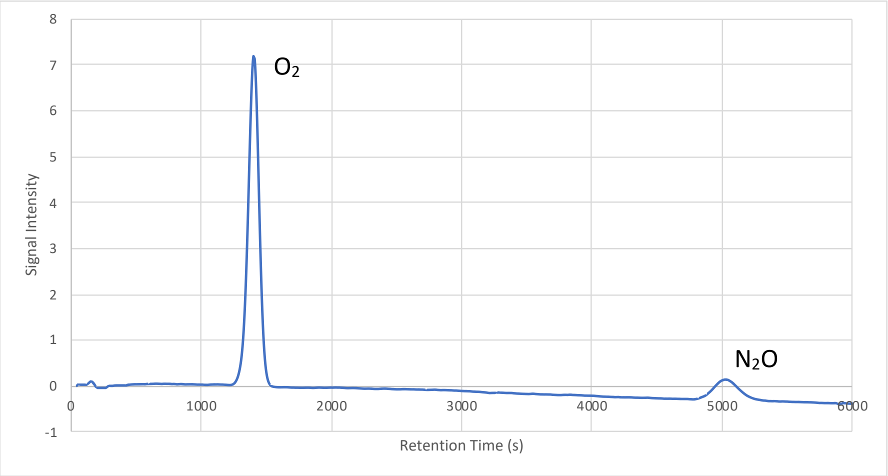

Figure S18. Gas chromatography head space analysis of the reduction of tetrabutylammonium nitrite by 1 equiv **1** and 1 equiv PPh_3 showing formation of nitrous oxide. The presence of O_2 is due to atmospheric contamination during injection.

Crystal Structure of 2

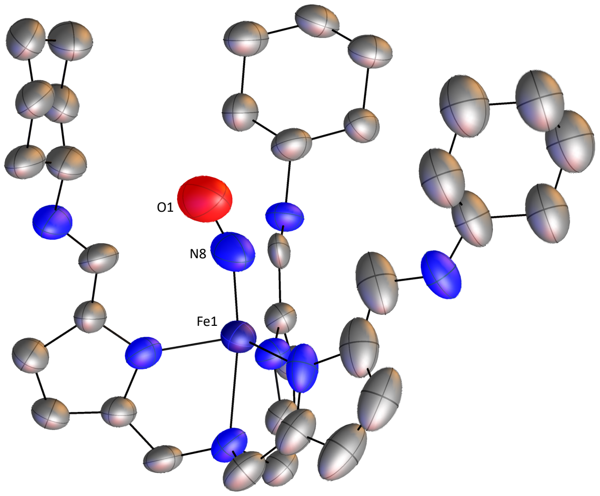

Figure S19. Thermal ellipsoids plot (50 % probability) of **2**. Hydrogen atoms are omitted for clarity. Triflate and THF molecules are not shown. Only major part of the disordered Fe complex shown.

Crystallographic Parameters of 2

Formula	$C_{41.2}H_{60.4}F_6FeN_8O_{7.8}S_2$
$D_{calc.}$/ g cm^{-3}	1.343
μ/mm^{-1}	3.814
Formula Weight	1026.54
Color	brown
Shape	needle-shaped
Size/mm^3	0.27×0.03×0.02
T/K	103.80
Crystal System	monoclinic
Space Group	$P2_1/c$
a/Å	15.6505(4)
b/Å	27.6628(8)
c/Å	12.1436(3)
α/°	90
β/°	105.002(2)
γ/°	90
V/Å^3	5078.2(2)
Z	4
Z'	1
Wavelength/Å	1.54178
Radiation type	CuK$_\alpha$
Θ_{min}/°	2.923
Θ_{max}/°	50.425
Measured Refl's.	18000
Indep't Refl's	5277
Refl's I≥2 σ(I)	3411
R_{int}	0.0980
Parameters	745
Restraints	1852
Largest Peak	0.998
Deepest Hole	-0.500
GooF	2.211
wR_2 (all data)	0.3695
wR_2	0.3355
R_1 (all data)	0.1918
R_1	0.1450

Computational Methods

The reaction mechanism of the reduction of the Fe-bound nitrosyl complex $[N(afa^{Cy})_3Fe(NO)]^{2+}$ (**2**) to yield ammonia and $[N(afa^{Cy})_2(pi^{Cy})Fe(OH)]$ (**4**) in the presence of potassium graphite (KC_8) as reductant and HOTf as proton source (Eq. 1) was investigated through density functional theory (DFT) calculations. Calculations were carried out using the dispersion-corrected hybrid exchange-correlation functional ωB97XD,[7] implemented in the Gaussian16 software.[8]

$$[N(afa^{Cy})_3Fe(NO)]^{2+} + 5e^- + 3H^+ \rightarrow [N(afa^{Cy})_2(pi^{Cy})Fe(OH)] + NH_3 \qquad (1)$$

The Fe atom was described with the Lanl2DZ effective core potential and its associated double-zeta basis set, along with an *f*-polarization function (exponent = 2.780).[9] C and H atoms were modelled with the double-zeta 6-31G(d, p) basis sets, while the more electronegative N, O, S, and F atoms were modeled using the 6-31+G(d,p) basis set. The Fe center was modelled in a high-spin state based on experimental magnetic data presented in this work and related literature.[10] For metal complexes involving radicals, both ferro- and anti-ferromagnetic coupling spin states were considered, *i.e.*, the radical's single electron having the same spin as the Fe metal center (S = 1/2 + 1/2) or opposite spins (S = 0). All possible conformers, with the tripodal ligand arms each facing in or out, were explicitly considered, but only the lowest energy conformers are discussed in this work.

Geometry optimizations were performed in vacuum without imposing any symmetry constraints. The nature of the stationary points was confirmed through vibrational frequency analysis, with energy minima displaying only real frequencies. The effect of the solvent used in experiments (THF, ε = 7.4257) was accounted for through single-point calculations with the SMD continuum solvation model. Additionally, the H-bonding acceptor ability of THF was considered by adding an energy correction of –4.8 kcal/mol for each ligand arm with the "NH" unit facing outwards, capable of forming a H-bond interaction with the THF solvent.[11] Standard state corrections were applied by adding (subtracting) 1.90 kcal/mol to the computed Gibbs reaction energies for every additional molecule with respect to the products (reactants).[12]

To determine the relative Gibbs energies of the reaction intermediates involved in the reduction of nitric oxide, we adopted the approach of Van Voorhis *et al.*,[13] shown in Eq. 2. This approach is similar to the computational hydrogen electrode model method proposed by Nørskov *et al*.[14] This method has been applied to study similar proton transfer (PT), electron transfer (ET) and proton-coupled electron transfer (PCET) processes previously, yielding notably satisfactory results.[15, 16]

$$\Delta G_{THF} = \Delta G_{THF}^{EA} + n_{H+} \times \Delta G_{(aq)}^{H+} + n_e \times (U + 4.28\ V) \qquad (2)$$

Here the ΔG_{THF}^{EA} is the Gibbs energy difference between the reactants and products, excluding the energies of the transferred proton and electron. $\Delta G_{(aq)}^{H+}$ is the Gibbs energy of solvation for a proton in aqueous solution. Given the high acidity of triflic acid, we adopted the widely accepted value of –11.803 eV.[13] The number of protons and electrons involved in the elementary steps are represented as n_{H+} and n_e, respectively. The U denotes the applied potential, which in this work corresponds to the standard reduction potential of the chemical reductant, KC_8, which is –2.04 V versus the standard hydrogen electrode (SHE). The 4.28 V represents the absolute value of the SHE, as taken from the literature.[17] The method applied to determine this potential uses the absolute solvation free energy of a proton, calculated using the cluster pair approximation method. Enthalpy and entropy corrections are then added by numerically solving equations from Fermi-Dirac statistics.[17] This method has been shown to give consistent and sufficiently accurate agreement with experimental data.

Conformers of Compound 2

Scheme S1. Modeled conformers of compound **2** obtained by rotating the N(afa^{Cy}) ligand arms. The conformers are labeled based on the number of N atoms in *cis* position within the same ligand arm (*i.e.* 0 *cis*, 1 *cis*, 2 *cis*, and 3 *cis*). Their relative Gibbs energies (in kcal/mol) are provided in bold. Mulliken spin densities (in a.u.) for the Fe, N, and O atoms in each conformer are shown, along with the Fe–N–O bond angle. The DFT-optimized structures for each conformer are displayed below, with relevant bond distances reported in Å.

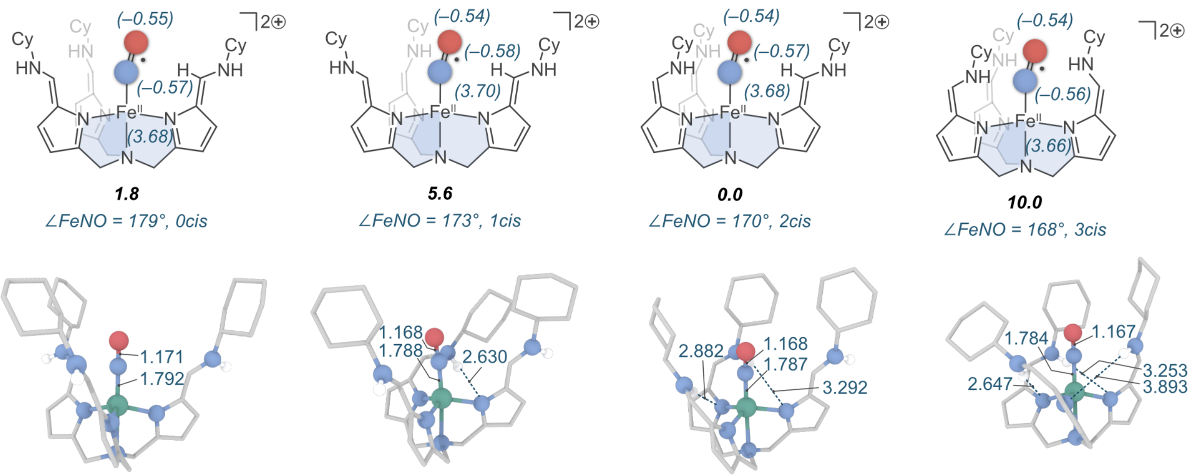

Multiconfigurational CASSCF and CASCI Methods

This section provides a detailed outline of the computational approach employed to analyze the oxidation state and electronic structure of complex **2**, serving as a guide for similar investigations in systems with multireference character. Similar computational analyses have been conducted on NO-bound complexes of Fe, Co, Ni, and Cu to elucidate their electronic structures.[18,19]

The geometry of complex **2**, optimized using unrestricted DFT in the quartet spin state, revealed antiferromagnetically coupling between the Fe and NO moieties, as indicated by the Mulliken spin densities of +3.68, –0.57, and –0.54 a.u. on Fe, N, and O atoms, respectively. The Fe spin, lying in between that of a high-spin Fe(II) (4 unpaired electrons) and intermediate-spin Fe(III) (3 unpaired electrons), introduced ambiguity regarding the precise oxidation state (Figure S14a). However, the total spin density on NO exceeding –1.00 (*i.e.* –1.11 a.u.) suggests a diradical character, as seen in similar Tp*M(NO) complexes (Tp* = hydro-tris(3,5-Me_2-pyrazolyl)borate; M = Co, Ni).[19] Thus, we inferred a greater Fe(III)(*S*=3/2)–NO^-(S=1) character rather than Fe(II)(*S*=2)–NO^0(*S*=1/2). Furthermore, the broken-symmetry DFT-optimized structure of **2** showed notable spin-contamination ($\langle S^2 \rangle = 4.84$), exceeding the expected theoretical value of 3.75 by 28.9%, suggesting a multideterminant ground state wave function. To address this, we applied multiconfigurational self-consistent field methods to confirm the Fe oxidation state and obtain a more accurate picture of its electronic structure.

Using Gaussian 16, we performed complete active space self-consistent field (CASSCF)[20] calculations on the quartet DFT-optimized structure of complex **2** via single-point calculations. We generated unrestricted natural orbitals (UNOs) with the "Pop=NaturalOrbitals" keyword at the UHF/STO-3G level, examining these orbitals to define a CAS(7,7) active space. This space, comprising 7 electrons and 7 orbitals, captures Fe-*d* and NO-π^* interactions, including bonding (BD) and antibonding (BD*) combinations of Fe-$d_{xy/yz}$ and NO-π^* orbitals, as well Fe-*d* based singly occupied molecular orbitals (SOMOs) (d_{z^2}, $d_{x^2-y^2}$, and d_{xz}).

To generate the initial CAS orbitals, a CASSCF(7,7,UNO) calculation was performed with the minimal STO-3G basis set, facilitating the convergence of molecular orbitals (MOs) and configuration interaction (CI) coefficients.[21] We then used these results as initial guesses, incrementally increasing the basis set from 3-21G to 6-31G. The optimized CAS MOs and their occupancies are presented in Figure S14b. Among the most populated orbitals in the active space are two bonding (d+π^*) orbitals, with occupancies of 1.49 and 1.48, comprising approximately 59% Fe-*d* and 39% NO-π^* character. The three non-bonding SOMOs maintained occupancies near 1 and are mainly Fe-*d* orbitals (>93%). Notably, the least populated anti-bonding (d+π^*) MOs, with occupancies of around 0.5, align with the DFT-predicted Mulliken spin densities of –0.57 and –0.54 a.u. on N and O atoms, supporting NO^-'s diradical character (*S* = 1).[18,19] However, a valence bond-like interpretation could not be assigned due to the delocalized nature of the CASSCF orbitals, preventing classification of the BD and BD* orbitals as either Fe or NO-centred.

To overcome this issue, we localized the CAS orbitals using Pipek-Mezey localization[22] with the MOKIT Python package.[23] Figure S14c illustrates these localized orbitals. We then performed a CASCI(7,7) calculation on the localized orbitals using ORCA 5.0,[24] maintaining the same active space as in the CASSCF(7,7) calculations. Table S1 summarizes the resulting configurations and their weights. Analysis of the CASCI wave functions reveals that Fe(III) was present in the *S* = 3/2 intermediate-spin state in 82.75% of the configurations and in the *S* = 5/2 high-spin state in 7.58%, correlating with the DFT-derived Mulliken spin density on Fe (+3.68 a.u.), which is above 3 for *S* = 3/2 but below 5 for *S* = 5/2. These findings, discussed further in the main text, identify the Fe(III)(*S*=3/2)–NO^-(*S*=1) configuration as the most accurate description for the electronic structure of complex **2**.

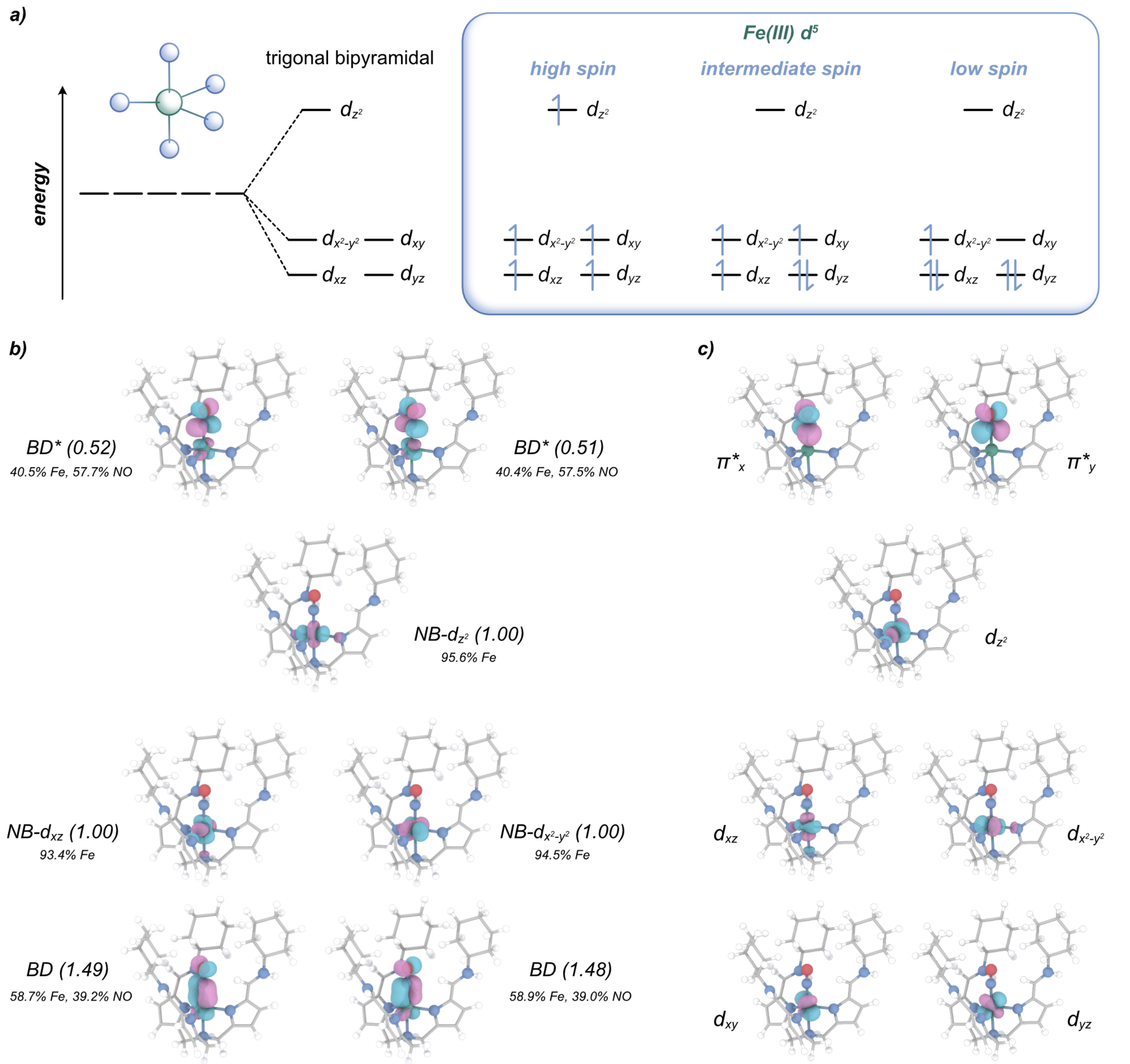

Figure S20. a) Crystal field splitting diagram of the transition metal *d* orbitals in a trigonal bipyramidal coordination environment, depicting the high, intermediate, and low spin states of Fe(III) within this environment. b) CAS orbitals (CASSCF) for complex **2** before localization. The bonding (BD), anti-bonding (BD*), and non-bonding (NB) nature of the orbitals are depicted, along with the percentage contributions of Fe-*d* and NO-*π** orbitals. c) Localized CAS orbitals (CASCI) obtained after Pipek-Mezey localization, labeled with the valence bond descriptions.

Table S1. Pipek-Mezey localized orbital-based wave function configurations for complex **2**, obtained from the CASCI(7,7) ground state calculations. Only configurations with a weight greater than 1% are included.

Configuration weight (%)	Orbital occupancies for configurations with > 1% weight						
	π^*_x	π^*_Y	d_{z^2}	d_{xz}	$d_{x^2-y^2}$	d_{xy}	d_{yz}
23.09	1	1	1	1	2	1	0
12.35	0	2	1	1	2	1	0
10.05	1	1	1	1	2	0	1
9.89	2	0	1	1	2	1	0
6.07	1	1	1	2	1	1	0
5.44	0	2	1	1	2	0	1
4.25	2	0	1	1	2	0	1
3.87	1	1	1	1	1	1	1
3.25	0	2	1	2	1	1	0
2.79	1	1	1	0	2	1	1
2.59	2	0	1	2	1	1	0
2.03	0	2	1	1	1	1	1
1.68	2	0	1	1	1	1	1
1.55	0	2	1	0	2	1	1
1.15	2	0	1	0	2	1	1

Alternative Reaction Pathways for Ammonia Formation from Complex 2

Scheme S2. Alternative pathways involving only intermolecular proton transfers (pathway B), considered for the a) reduction of intermediate **I3**, b) oxidation of the metal center in intermediate **I5**, and c) reduction of intermediate **I5** to ammonia and compound **4**. The relative Gibbs energies for each complex are calculated in THF at 298 K and 1 atm, reported in eV. The N(afa^{Cy}) and N(pi^{Cy}) ligand arms are shaded in blue and red, respectively. The Gibbs energies for each step are indicated along the reaction arrows.

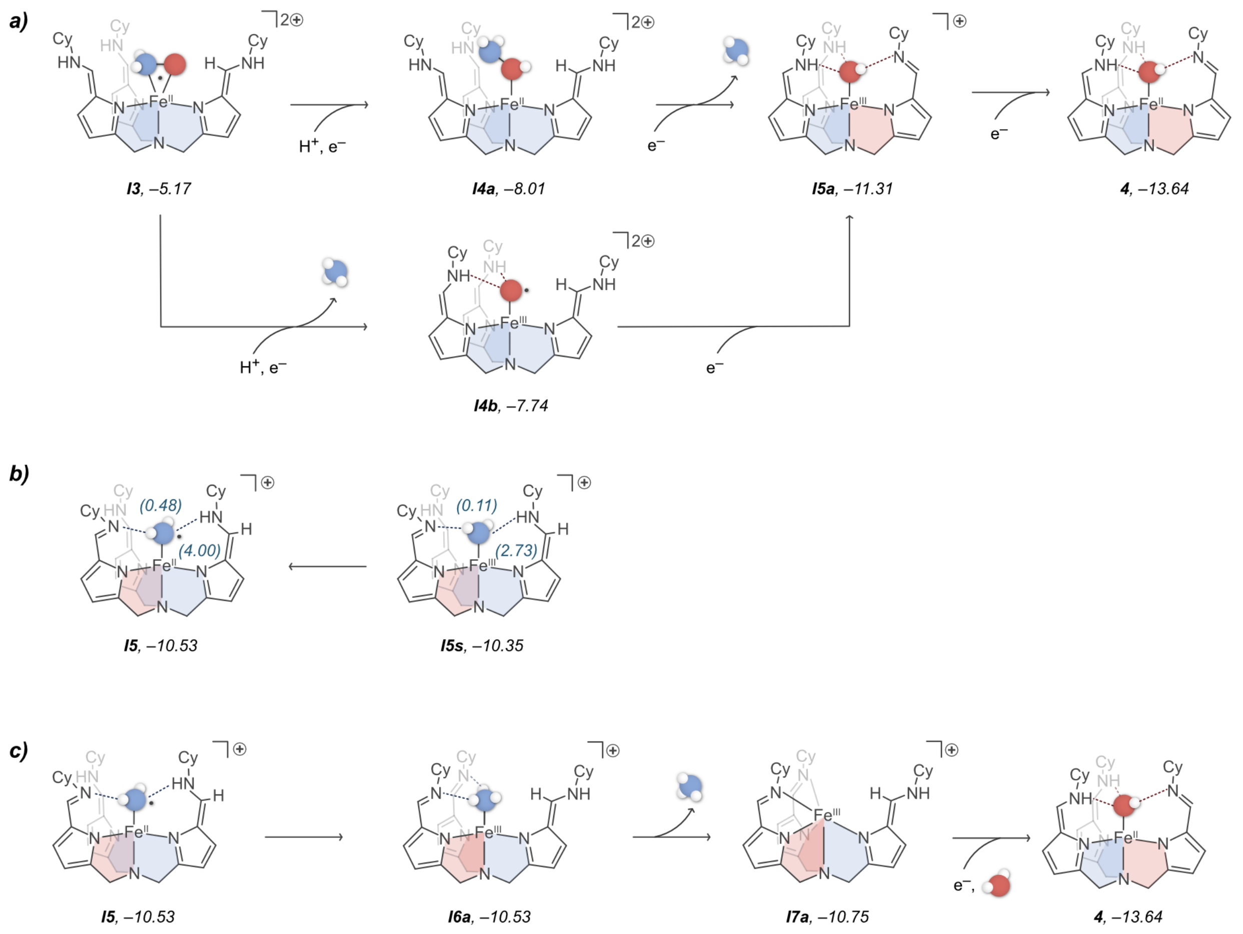

Scheme S3. Alternative pathways involving only intramolecular proton transfers (pathway A), considered for a) the reduction of intermediate **I4'** to **I5'**, and b) the reduction of intermediate **I3'** to NH_3 and complex **4**. The Gibbs energies (in eV) associated with each reaction step are calculated in THF at 298 K and 1 atm. The N(afa^{Cy}) and N(pi^{Cy}) ligand arms are shaded in blue and red, respectively.

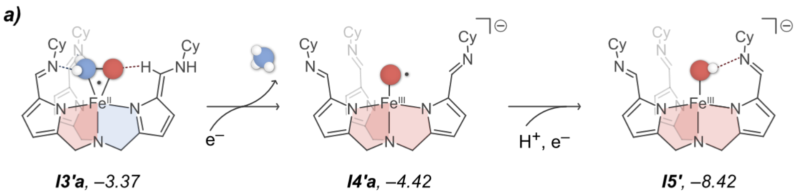

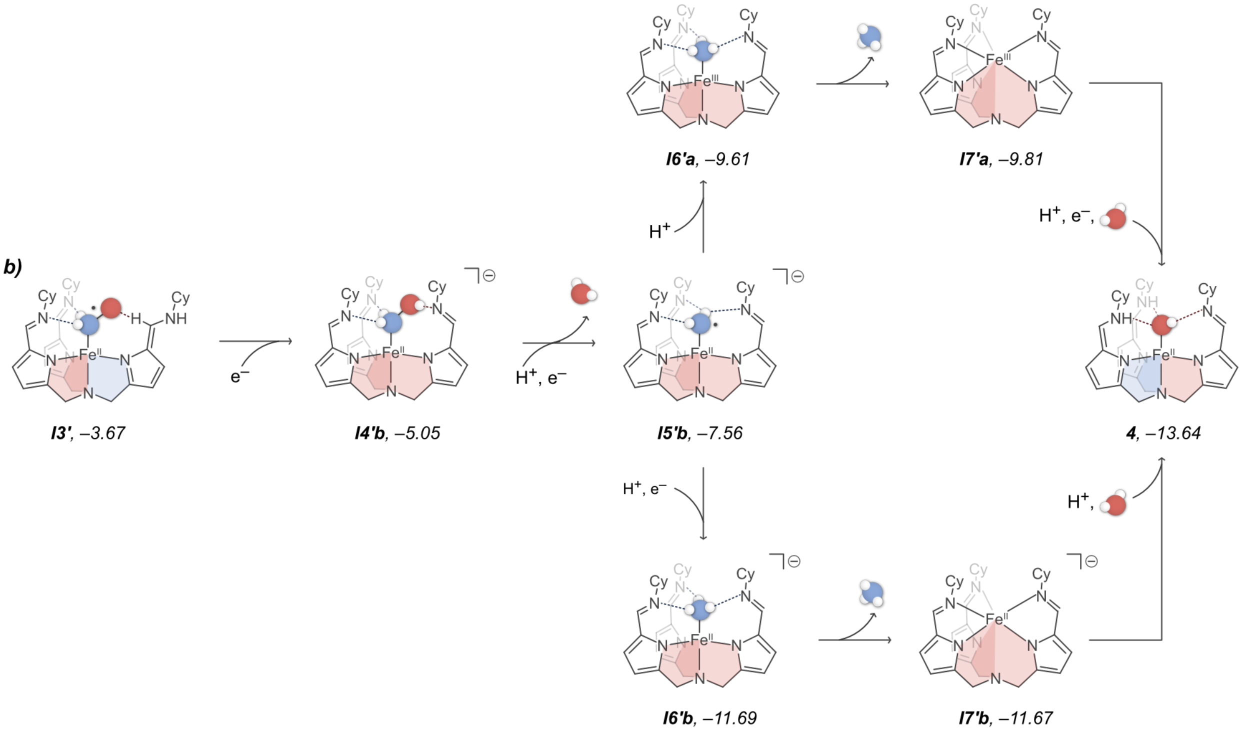

Non-Covalent Interaction (NCI) analysis

NCI analysis was performed using the Critic2 software.[25,26] Below, we provide a brief overview of the theory behind NCI analysis. For a more detailed description, please refer to the original article by Contreras *et al*.[27] NCI analysis has been widely applied to both molecular and periodic systems yielding satisfactory results.[28]

NCI analysis examines the correlation between the reduced density gradient (RDG) and a function of the electron density (ρ), which can be used to distinguish the types of NCIs present in a chemical system. The RDG characterizes the electron density regions where the reduced density matrix tends to zero. At these regions, the RDG also approaches zero, indicating the presence of an inflection point between two density maxima. The RDG can be expressed as a function of the gradient of ρ as:

$$s(r) = \frac{1}{2(3\pi^2)^{\frac{1}{3}}} \frac{|\nabla\rho(r)|}{\rho(r)^{\frac{4}{3}}} \tag{3}$$

The strength of the NCIs is proportional to the 'actual electron density' at these points, while the attractive or repulsive nature of the interaction can be assessed by investigating the curvature of the electron density ($\nabla^2\rho$). The latter is dominated by attractive electron-core interactions. Therefore, the local curvatures in the diagonalized Hessian of the electronic density matrix (which contains the second-order partial derivatives) are better indicators, particularly the second eigenvalue (λ_2). Specifically, λ_2 is negative (positive) for attractive (repulsive) interactions. Thus, a plot of the RDG (y axis) against sign(λ_2)ρ (x axis) allows us to identify NCIs as peaks in the electron density where the RDG is zero.

Cartesian Coordinates and Energies of DFT-Modeled Structures

All the DFT data underlying this work, including the cartesian coordinates and energies of all the modelled structures, is free and openly accessible via the following ioChem-BD online dataset:

DOI: 10.19061/iochem-bd-6-402

References

(1) K. S. Hagen, *Inorg. Chem.* **2000**, *39*, 5867–5869.

(2) E. M. Matson, Y. J. Park, A. R. Fout, *J. Am. Chem. Soc.* **2014**, *136*, 17398–17401.

(3) E. M. Matson, J. A. Bertke, A. R. Fout, *Inorg. Chem.* **2014**, *53*, 4450–4458.

(4) J. J. Curley, R. G. Bergman, T. D. Tilley, *Dalton Trans.* **2012**, *41*, 192–200.

(5) C. L. Ford, Y. J. Park, E. M. Matson, Z. Gordon, Z. A. R. Fout, *Science,* **2016**, *354*, 741–743.

(6) D. Scheiner, Water Res. **1975**, *10*, 31–36.

(7) J.-D. Chai, M. Head-Gordon, *Phys. Chem. Chem. Phys.* **2008**, *10*, 6615–6620.

(8) Gaussian 16, Revision C.01, M. J. Frisch, G. W. Trucks, H. B. Schlegel, G. E. Scuseria, M. A. Robb, J. R. Cheeseman, G. Scalmani, V. Barone, G. A. Petersson, H. Nakatsuji, X. Li, M. Caricato, A. V. Marenich, J. Bloino, B. G. Janesko, R. Gomperts, B. Mennucci, H. P. Hratchian, J. V. Ortiz, A. F. Izmaylov, J. L. Sonnenberg, D. Williams-Young, F. Ding, F. Lipparini, F. Egidi, J. Goings, B. Peng, A. Petrone, T. Henderson, D. Ranasinghe, V. G. Zakrzewski, J. Gao, N. Rega, G. Zheng, W. Liang, M. Hada, M. Ehara, K. Toyota, R. Fukuda, J. Hasegawa, M. Ishida, T. Nakajima, Y. Honda, O. Kitao, H. Nakai, T. Vreven, K. Throssell, J. A. Montgomery, Jr., J. E. Peralta, F. Ogliaro, M. J. Bearpark, J. J. Heyd, E. N. Brothers, K. N. Kudin, V. N. Staroverov, T. A. Keith, R. Kobayashi, J. Normand, K. Raghavachari, A. P. Rendell, J. C. Burant, S. S. Iyengar, J. Tomasi, M. Cossi, J. M. Millam, M. Klene, C. Adamo, R. Cammi, J. W. Ochterski, R. L. Martin, K. Morokuma, O. Farkas, J. B. Foresman, and D. J. Fox, Gaussian, Inc., Wallingford CT, **2016**.

(9) A. W. Ehlers, M. Böhme, S. Dapprich, A. Gobbi, A. Höllwarth, V. Jonas, K. F. Köhler, R. Stegmann, A. Veldkamp, G. Frenking, *Chem. Phys. Lett.* **1993**, *208*, 111–114.

(10) a) Y. J. Park, M. N. Peñas-DeFrutos, M. J. Drummond, Z. Gordon, O.R. Kelly, M. García-Melchor, A. R. Fout, *Inorg. Chem.* **2022**, *61*, 8182–8192; b) C. L. Ford, Y. J. Park, E. M. Matson, Z. Gordon, A. R. Fout, *Science*, **2016**, *354*, 741–743.

(11) A. V. Marenich, C. J. Cramer, D. G. Truhlar, *J. Phys. Chem. B,* **2009**, *113*, 6378–6396.

(12) J. H. Jensen, *Phys. Chem. Chem. Phys.* **2015**, 17, 12441–12451.

(13) L-P. Wang, Q. Wu, T. V. Voorhis, *Inorg. Chem.* **2010**, *49*, 4543–4553.

(14) J. K. Nørskov, J. Rossmeisl, A. Logadottir, L. Lindqvist, J. R. Kitchin, T. Bligaard, and H. Jónsson, *J. Phys. Chem. B,* **2004**, *108*, 17886–17892.

(15) A. M. Ako, A. C. Kathalikkattil, R. Elliott, J. Soriano-López, I. M. McKeogh, M. Zubair, B. Twamley, N. Zhu, M. García-Melchor, P. E. Kruger, W. Schmitt, *Inorg. Chem.* **2020**, *59*, 14432–14438.

(16) M. J. Craig, G. O. Coulter, E. T. Dolan, J. Soriano-López, E. Mates-Torres, W. Schmitt, M. García-Melchor, *Nat. Commun.* **2019**, *10*, 4993.

(17) P. Miró, M. Z. Ertem, L. Gagliardi, C. J. Cramer, *Molecular Water Oxidation Catalysis*, editor Llobet, A., John Wiley & Sons, Ltd, **2014** 233–255.

(18) M. Radoń, E. Broclawik, K. Pierloot, *J. Phys. Chem. B,* **2010**, *114*, 1518–1528.

(19) N. C. Tomson, M. R. Crimmin, T. Petrenko, L. E. Rosebrugh, S. Sproules, W. C. Boyd, R. G. Bergman, S. DeBeer, D. Toste, K. Wieghardt, *J. Am. Chem. Soc.* **2011**, *133*, 18785–18801.

(20) M. Klene, M. A. Robb, M. J. Frisch, and P. Celani, *J. Chem. Phys.*, **2000**, *113*, 5653–5665.

(21) J. M. Bofill, P. Pulay, *J. Chem. Phys.*, **1989**, *90*, 3637–3646.

(22) J. Pipek, P. G. Mezey, *J. Chem. Phys.* **1989**, *90* (1989) 4916–4926.

(23) J. Zou, MOKIT program, https://gitlab.com/jxzou/mokit (accessed Apr 13, 2024).

(24) a) F. Neese, The ORCA program system Wiley Interdiscip. Rev.: *Comput. Mol. Sci.* **2012**, *2*, 73–78; b) F. Neese, Software update: the ORCA program system -- Version 5.0 Wiley Interdiscip. Rev.: *Comput. Mol. Sci.* **2022**, *12*, e1606.

(25) A. Otero-de-la-Roza, E. R. Johnson, V. Luaña, *Comput. Phys. Commun*. **2014**, *185*, 1007–1018.

(26) A. Otero-de-la-Roza, M. A. Blanco, A. M. Pendás, V. Luaña, *Comput. Phys. Commun.* **2009**, *180*, 157–166.

(27) J. Contreras-García, E. R. Johnson, S. Keinan, R. Chaudret, J.-P. Piquemal, D. N. Beratan, W. Yang, *J. Chem. Theory Comput*. **2011**, *7*, 625−632.

(28) a) D. Kehoe, E. Mates-Torres, P. Samokhvalov, M. García-Melchor, Y. K. Gun'ko, *J. Phys. Chem. C*. **2021**, *126*, 434–443; b) C. Sahm, E. Mates-Torres, N. Eliasson, K. Sokolowski, A. Wagner, K. Dalle, Z. Huang, O. Scherman, L. Hammarström, M. García-Melchor, E. Reisner, *Chem. Sci.* **2021**, *12*, 9078–9087; c) C. D. Sahm, A. Ciotti, E. Mates-Torres, V. Badiani, K. Sokołowski, G. Neri, A. J. Cowan, M. García-Melchor, E. Reisner, *Chem. Sci*. **2022**, *13*, 5988–5998; d) D. C. Nájera, M. N. Peñas-Defrutos, M. García-Melchor, A. R. Fout, *Chem. Commun.* **2022**, *58*, 9626-9629.
